# Increased ParB level affects expression of stress response, adaptation and virulence operons and potentiates repression of promoters adjacent to the high affinity binding sites *parS3* and *parS4* in *Pseudomonas aeruginosa*

**DOI:** 10.1371/journal.pone.0181726

**Published:** 2017-07-21

**Authors:** Adam Kawalek, Krzysztof Glabski, Aneta Agnieszka Bartosik, Anna Fogtman, Grazyna Jagura-Burdzy

**Affiliations:** 1 Institute of Biochemistry and Biophysics, Polish Academy of Sciences, Department of Microbial Biochemistry, Warsaw, Poland; 2 Institute of Biochemistry and Biophysics, Polish Academy of Sciences, Laboratory of Microarray Analysis, Warsaw, Poland; University of Oklahoma, UNITED STATES

## Abstract

Similarly to its homologs in other bacteria, *Pseudomonas aeruginosa* partitioning protein ParB facilitates segregation of newly replicated chromosomes. Lack of ParB is not lethal but results in increased frequency of anucleate cells production, longer division time, cell elongation, altered colony morphology and defective swarming and swimming motility. Unlike in other bacteria, inactivation of *parB* leads to major changes of the transcriptome, suggesting that, directly or indirectly, ParB plays a role in regulation of gene expression in this organism. ParB overproduction affects growth rate, cell division and motility in a similar way as ParB deficiency. To identify primary ParB targets, here we analysed the impact of a slight increase in ParB level on *P*. *aeruginosa* transcriptome. ParB excess, which does not cause changes in growth rate and chromosome segregation, significantly alters the expression of 176 loci. Most notably, the mRNA level of genes adjacent to high affinity ParB binding sites *parS1*-*4* close to *oriC* is reduced. Conversely, in cells lacking either *parB* or functional *parS* sequences the orfs adjacent to *parS3* and *parS4* are upregulated, indicating that direct ParB- *parS3/parS4* interactions repress the transcription in this region. In addition, increased ParB level brings about repression or activation of numerous genes including several transcriptional regulators involved in SOS response, virulence and adaptation. Overall, our data support the role of partitioning protein ParB as a transcriptional regulator in *Pseudomonas aeruginosa*.

## Introduction

Accurate copying and segregation of genetic material to progeny cells is crucial for survival and maintenance of species identity. The process of bacterial DNA segregation was first deciphered for low-copy-number plasmids [1–4]. Active partitioning of such plasmids during cell division requires three specific plasmid-encoded factors: an NTPase (A-component), a DNA-binding protein (DBP, B-component) and a special site in DNA, designated centromere-like sequence (*parS*/*parC*). Interactions of DBP with *parS* sequence(s) lead to formation of segrosomes which are then separated by a dynamic NTPase machinery to polar positions assuring their proper segregation during the subsequent cell division [2,3]. Plasmidic active partition systems have been classified into three groups based on the type of NTPase and structure of DBP [2,4]. Homologs of plasmidic Type IA partition proteins, Walker-type ATPases (ParAs) and large DBPs with helix-turn-helix motifs (ParBs), which after binding to *parS* spread on DNA and form large nucleoprotein complexes [5], are also encoded on the majority of bacterial chromosomes [3,4,6]. Multiple copies of highly conserved *parS* sequences are mainly clustered in the so-called *ori* domain comprising ca. 20% of the chromosome [7].

The role of the ParABS systems in accurate bacterial chromosome segregation is widely acknowledged but varies from essential, as exemplified by *Caulobacter crescentus* [8] or *Myxococcus xanthus* [9], to accessory, as in *Bacillus subtilis* [10–13], *Streptomyces coelicolor* [14], *Vibrio cholerae* [15,16] or *Pseudomonas aeruginosa* [17–19]. Apart from their well-established role in the segregation of newly replicated *ori* domains through DNA compaction [20–22], proper positioning of *ori* domains in the cell [19,23], Par proteins have also been shown to play a role in the control of DnaA activity and replication initiation [24,25] as well as in coordination of cell cycle and differentiation [26–28]. Chromosomal ParB homologs bind to *parS in vitro*, polymerize on DNA and bridge distant sequences [13,29–32]. Whole-genome analyses using chromatin immunoprecipitation (ChIP) have demonstrated spreading of ParB homologs around *parS* sites for up to 20 kb [29,33,34]. Despite the ParB spreading, a transcriptomic analysis in *B*. *subtilis* did not identify any significant changes in gene expression in a *spo0J* null mutant (Spo0J is a ParB homolog) relative to a WT strain [34]. Limited ParB-dependent transcriptional silencing in the proximity of *parS* sequences has been observed only for several genes in *V*. *cholerae* [35] and *S*. *pneumoniae* [36].

In *P*. *aeruginosa* a lack of ParA and/or ParB is not lethal but results in up to 1000-fold increased frequency of production of anucleate cells even during growth in optimal conditions [17,18,37]. Various *par* mutants exhibit longer division time, increase in cell size, altered colony morphology and are impaired in swarming and swimming motility [17,18]. Ten *parS* sites scattered in the chromosome of *P*. *aeruginosa* have been identified, but only four of them closest to *oriC* seem to be involved in chromosome segregation [33,38]. A transcriptomic analysis of *P*. *aeruginosa parA* and *parB* mutants has demonstrated changes in expression of hundreds of loci [39], including genes related to stress response but also many known and putative transcriptional regulators, suggesting a direct and/or indirect role of Par proteins in the regulation of gene expression. In test plasmids, ParB of *P*. *aeruginosa* was found to spread around *parS* and silence nearby promoters [37], but a comparison of the *parB* mutant and WT transcriptomes did not reveal any obvious changes in the expression of genes adjacent to chromosomal *parS* sequences [39]. However, a recent ChIP-seq analysis of ParB distribution on the *P*. *aeruginosa* chromosome has revealed, in addition to the region around the high affinity *parS1-4* sequences, also secondary ParB binding sites, apparently not related to known *parS* sites [33].

Similarly to the lack of ParB, also its excess in *P*. *aeruginosa* affects the cell cycle, as highlighted by slower growth rate, and causes cell elongation and defects in swarming and swimming. ParB excess manifests in nucleoid condensation and increased frequency of anucleate cells formation [17,37,40,41]. The toxicity of ParB overproduction is significantly diminished when ParB is impaired in its polymerization domain [40] whereas ParB variants defective in interactions with DNA, either due to the inability to form dimers or mutations in the DNA binding domain, demonstrate no toxicity [40,41]. This suggests that DNA binding and spreading of ParB protein is crucial for its biological function.

In this project we analysed changes in the transcriptome of *P*. *aeruginosa* cells with a slightly increased ParB level, not affecting growth rate and chromosome segregation, to define the primary targets of ParB.

## Materials and methods

### Bacterial strains and growth conditions

Bacterial strains used in this study are listed in Table 1. Cultures were grown in L broth [42] or on L agar (L broth solidified with 1.5% agar) at 37^°^C. For selection of plasmids *Escherichia coli* media were supplemented with 150 μg ml^-1^ (liquid cultures) or 300 μg ml^-1^ (solid media) penicillin (Pn), 10 µg ml^-1^ chloramphenicol (Cm) or 50 μg ml^-1^ kanamycin (Km). Chloramphenicol at concentration 75 μg ml^-1^ (liquid cultures) and 150 μg ml^-1^ (solid media) was used to maintain plasmids in *P*. *aeruginosa*. 300 μg ml^-1^ carbenicillin (Cb) and 300 μg ml^-1^ rifampicin (Rif) was added to the solid medium for the selection of *P*. *aeruginosa* transconjugants in allele exchange procedure. To induce the expression from the *araBAD* promoter arabinose (Ara) was added to 0.02% to cultures of PAO1161 with pKGB8 (*araBAD*p) or pKGB9 (*araBAD*p-*parB*) and to 0.1% in PAO1161::*araBAD*p or PAO1161::*araBAD*p-*flag-parB* cultures. For MIC analysis of PAO1161 (pKGB8) and PAO1161 (pKGB9) and PAO1161 as a control, strains were grown in Mueller Hinton cation adjusted medium in the range of Ara concentrations (0, 0.02 and 0.2%) and in the presence of a gradient of piperacillin, ticarcillin, ciprofloxacin, gentamicin, tobramycin or imipenem.

**Table 1:** Bacterial strains used in this study.

Strain	Description	Reference
***Escherichia coli***		
DH5α	F^–^ Φ80*lacZ*Δ*M15* Δ(*lacZYA-argF) U169 recA1 endA1 hsdR17* (r_K–_, m_K+_) *phoA supE44 λ thi-1 gyrA96 relA1*	[43]
S17-1	*pro* Δ*hsdR hsdM*^+^ *recA* Tp^R^ Sm^R^ ΩRP4-Tc::Mu Kn::Tn7	[44]
***Pseudomonas aeruginosa***		
PAO1161	PAO1161 Rif^R^ *leu*	[17]
PAO1161 *parB*_*null*_	PAO1161 Rif^R^ *leu parB*1-18::*tetM*, Tc^R^	[17]
PAO1161 *parS*_null_	PAO1161 Rif^R^ with ten *parS* sequences mutated or deleted to impair ParB binding to these sequences	[38]
PAO1161::*araBAD*p	PAO1161 Rif^R^ with *araC araBAD*p T_*rrnB*_^1^ cassette integrated in intergenic region between *PA5412* and *PA5413* (at position 6091513 nt)	this study
PAO1161::*araBAD*p-*flag*-*parB*	PAO1161 Rif^R^ with *araC araBAD*p-*flag*-*parB* T_*rrnB*_ cassette integrated in intergenic region between *PA5412* and *PA5413* (at position 6091513 nt)	this study
PAO1161::*bexR*p-*lacZ*	PAO1161 Rif^R^ Cb^R^ with *bexR*p-*lacZ* cassette integrated in intergenic region between *PA5412* and *PA5413* (at position 6091513 nt)	this study

^1^T_*rrnB*_ transcriptional terminator of *E*. *coli rrnB* gene

### Transformation and conjugation procedures

Transformation of *E*. *coli* and *P*. *aeruginosa* was performed by standard procedures [43,45]. Conjugation between transformants of *E*. *coli* S17-1 and *P*. *aeruginosa* PAO1161 Rif^R^ was performed on solid media as described previously [17].

### Plasmids and DNA manipulations

Plasmids used in this study are listed in Table 2. Plasmid manipulations were carried out by standard procedures [46]. Oligonucleotides used in this work are listed in S1 Table. All new plasmid constructs were verified by sequencing at the Laboratory of DNA Sequencing and Oligonucleotide Synthesis, IBB PAS.

**Table 2:** Plasmids used in this study.

Plasmid	Description	Reference
pABB1.0	pBBRMCS1 derivative, BHR^1^, *ori*_IncA/C_, Cm^R^	[47]
pABB28.2	pET28a with *his*-tag replaced by *flag*-tag, Km^R^	this study
pAKE600	suicide vector, *oriV*_MB1_, *sacB* gene, Pn^R^	[48]
pBAD24	expression vector, *oriV*_MB1_, *araC araBAD*p T_*rrnB*_ cassette, Pn^R^	[49]
pCM132	BHR^1^, dual replicon promoter-probe vector *oriV*_ColE1_, *oriV*_RK2_, *trfA*_RK2_, *oriT*_RK2_, promoter-less *lacZ*, Km^R^	[50]
pET28a	expression vector, *oriV*_MB1_, T7p, 6xHis-tag, Km^R^	Novagen, Inc.
pKAB132	pPJB132 with deletion of EcoRI-BglII fragment, control vector, Cm^R^, Km^R^	this study
pKAB133	pPJB132 with *PA0011*p-*lacZ*	this study
pKAB134	pPJB132 with PA0011p_*parS3*mut_-*lacZ*	this study
pKAB135	pPJB132 with *PA0013*p-*lacZ*	this study
pKAB136	pPJB132 with *PA0013*p_*parS4*mut_-*lacZ*	this study
pKAB240	pBAD24 with NsiI restriction site between T_*rrnB*_ and *bla*p	this study
pKAB241	pKAB240 with *flag* tagged *P*. *aeruginosa parB* (*araBAD*p*-flag-parB*)	this study
pKAB242	pKAB240 with T_*rrnB*_-*mcs-lacZ*(partial)*-*T_T7_^2^ cassette inserted as NsiI fragment	this study
pKAB243	pKAB242 with insertion of full-length *lacZ* and *mcs*, contains complete T_*rrnB*_-*mcs-lacZ-*T_T7_ cassette (NsiI fragment)	this study
pKAB600	pAKE600 with *P*. *aeruginosa* genomic fragment (coordinates 6090756–6092192) with PstI at position 6091513 nt	this study
pKAB601	pKAB600 with *araC araBAD*p T_*rrnB*_ cassette	this study
pKAB602	pKAB600 with *araC araBAD*p*-flag-parB* T_*rrnB*_ cassette	this study
pKAB603	pKAB600 with deletion of EcoRI restriction site	this study
pKAB604	pKAB603 with T_*rrnB*_-*mcs-lacZ-*T_T7_ cassette	this study
pKAB605	pKAB604 with *BexR*p cloned in the *mcs*, T_*rrnB*_-*BexR*p*-lacZ-*T_T7_ cassette	this study
pKGB8	pABB1.0 with *araC araBAD*p, expression vector	this study
pKGB9	pKGB8 with *araBAD*p-*parB*_*P*.*a*._	this study
pKRP10	*ori*_MB1_, contains Cm^R^ cassette, Pn^R^	[51]
pMKB5.2	*oriV*_MB1_, *cyaA*T18-*parB*_*P*.*a*_ translational fusion, Pn^R^	lab collection
pPJB132	pCM132 with Cm^R^ cassette, Cm^R^, Km^R^	this study

^1^ BHR, broad-host-range plasmid

^2^ T_T7_, T7 phage transcriptional terminator

Plasmid pKGB8 was constructed by ligation of Eco47III-PstI fragment from pBAD24 containing *araC araBAD*p with HincII and PstI digested pABB1.0. Subsequently, EcoRI-SacI fragment from pMKB5.2 containing *parB* ORF was cloned into pKGB8 to yield plasmid pKGB9.

pET28a (Novagen, Inc.) was modified to add *flag*-tag instead of *his*-tag to the coding sequences. Oligonucleotides #1 and #2 were combined, heated to 100^°^C for 5 minutes and left for annealing at room temperature. pET28a vector was digested using NcoI and NdeI and ligated with obtained synthetic DNA fragment to yield pABB28.2.

To facilitate transfer of the *araC-araBAD*p-T_*rrnB*_ cassette, an additional NsiI restriction site was introduced into pBAD24 downstream of T_*rrnB*_. Two fragments amplified by PCR using pBAD24 as a template and primer pairs #3/#4 and #5/#6, respectively, were used as a template in the second round of PCR with primers #3 and #6 to obtain a 1160 bp product that replaced the HindIII-ScaI fragment in pBAD24 to yield pKAB240.

The *flag*-*parB* fusion was constructed using similar strategy. The *flag* sequence amplified from pABB28.2 using primers #7/#8 and *P*. *aeruginosa parB* gene amplified from PAO1161 genomic DNA using primers #9/#10 were combined and used as a template in overlap PCR with primers #7/#10. The obtained PCR fragment with *flag-parB* fusion was digested with EcoRI and SalI and ligated into pKAB240 to yield pKAB241.

To construct PAO1161::*araBAD*p and PAO1161::*araBAD*p-*flag*-*parB* strains, the suicide plasmids pKAB601 and pKAB602 were constructed. First, the intergenic region between *PA5412* and *PA5413* from PAO1161 chromosome was amplified in two parts with pairs of primers #11/#12 and #13/#14. Primers #12 and #13 introduced a PstI site. The obtained PCR fragments were digested either with MfeI and PstI or PstI and BamHI, respectively, and the mixture was ligated with EcoRI and BamHI digested pAKE600 [48] to yield pKAB600. The *araC*-*araBAD*p-T_*rrnB*_ cassette from pKAB240 and *araC*-*araBAD*p-*flag-parB*-T_*rrnB*_ cassette from pKAB241 were excised as NsiI fragments and ligated into PstI site of pKAB600 to yield pKAB601 and pKAB602, respectively. *E*. *coli* S17-1 strain was transformed with pKAB601 or pKAB602 and the transformants were used as donor strains in conjugation with PAO1161 Rif^R^ [17]. The cassettes were integrated in the defined region of PAO1161 using the allele exchange procedure [17] to obtain PAO1161::*araBAD*p and PAO1161::*araBAD*p-*flag*-*parB*.

pCM132 was modified by insertion of Cm^R^ cassette (SphI fragment from pKRP10 [51]) into SphI site, downstream of *lacZ*, to yield pPJB132. Putative promoter sequences preceding *PA0011* and *PA0013* were amplified on PAO1161 genomic DNA using primer pairs #15/#16 and #17/#18, respectively. The *PA0011*p_*parS3*mut_ and *PA0013*p_*parS4*mut_ fragments were amplified on PAO1161 *parS*_null_ genomic DNA [38] as a template using the same primer pairs. PCR products were digested with EcoRI and BamHI and ligated between EcoRI and BglII sites into pPJB132 to yield plasmids pKAB133 (*PA0011*p-*lacZ*), pKAB134 (*PA0011*p_*parS3*mut_-*lacZ*), pKAB135 (*PA0013*p-*lacZ*) and pKAB136 (*PA0013*p_*parS4*mut_-*lacZ*). The empty control vector for β-galactosidase measurements (pKAB132) was obtained by digestion of pPJB132 with EcoRI and BglII, blunting by fill-in with Klenow fragment of DNA PolI and self-ligation.

To construct PAO1161 Rif^R^ strain with *bexR*p-*lacZ* cassette integrated in intergenic region between *PA5412* and *PA5413*, the suicide plasmid pKAB605 was constructed. First, the T_*rrnb*_ with *mcs*, part of the *lacZ* gene and T_T7_ was amplified using overlap PCR strategy. To this end, 2 fragments were amplified on pCM132 plasmid as a template using primer pairs #19/#20 and #21/#22, respectively, and the 3rd fragment was amplified on pET28a using primer pair #23/#24. The three fragments were mixed and used as a template in an overlap PCR with primers #19/#24. The obtained 787 bp product digested with NsiI replaced the *araC*-*araBAD*p-T_*rrnB*_ in pKAB240 yielding plasmid pKAB242. *mcs*-*lacZ* was subsequently excised from pCM132 as EcoRI and BlpI fragment and ligated with EcoRI-BlpI digested pKAB242 to yield pKAB243, containing complete T_*rrnB*_-*mcs-lacZ-*T_T7_ cassette.

The single EcoRI restriction site in pKAB600 plasmid was removed by EcoRI digestion, filling in by Klenow fragment of DNA PolI and plasmid self-ligation to yield pKAB603. The T_*rrnB*_-*mcs-lacZ-*T_T7_ cassette excised from pKAB243 by NsiI digestion was ligated into PstI site of pKAB603 to yield pKAB604. The region preceding *bexR* (*PA2432*) gene was amplified on PAO1161 genomic DNA using primers #25/#26, digested with EcoRI and BamHI and ligated with EcoRI and BglII digested pKAB604 to yield plasmid pKAB605 carrying T_*rrnB*_- *bexR*p*-lacZ-*T_T7_ cassette flanked by sequences allowing integration in the intergenic region between *PA5412* and *PA5413*. *E*. *coli* S17-1 strain transformed with pKAB605 was used as donor in conjugation with PAO1161 Rif^R^ [18]. The suicide vector was integrated in the defined region of PAO1161 (at position 6091513 nt) using homology recombination [18] and the carbenicillin-resistant integrant (PAO1161::*bexR*p-*lacZ*) was used as a recipient in a subsequent conjugation with S17-1 cells carrying pKGB8 or pKGB9 plasmids. Transconjugants were selected on L agar plates with carbenicillin, chloramphenicol and 40 μg ml^-1^ X-gal to allow visualization of *bexR*p-*lacZ* induction immediately after the introduction of plasmids.

### RNA isolation

*P*. *aeruginosa* strains taken from -80^°^C stocks were grown on L agar plates at 37^°^C and single colonies were used to inoculate three independent cultures (biological replicates). After overnight growth, the cultures were diluted 1:100 into fresh L broth and grown to the optical density 0.4–0.6 at 600 nm (OD_600_). RNA was isolated with RNeasy mini kit (Qiagen) according to the manufacturer’s protocol for bacterial cells from 2 ml of cultures mixed with 4 ml of RNAprotect Bacteria Reagent (Qiagen). Total RNA was digested with DNase (TURBO DNA-free Kit, Ambion) to remove genomic DNA.

### Microarray analysis

Microarray analysis was performed essentially as described before [39]. The raw microarray data have been deposited at the NCBI Gene Expression Omnibus database at accession number GSE95647 (release after publication acceptance). Gene expression data were analysed using Partek Genomic Suite v6.6 (Partek Inc., St. Louis, MO). Raw data were processed using GeneChip Robust Multiarray Averaging (GC RMA): background correction, quantile normalization, log2 transformation and median polish summarization. Analysis of variance (ANOVA) using REML (restricted maximum likelihood) was performed to identify differentially expressed genes. Gene lists were created using a cut-off of *p*-value≤0.05, with a fold change (FC) higher than 2 or lower than -2. Clustering of the genes according to expression pattern changes was performed by K-means clustering [52] using MultiExperiment Viewer v4.9 [53]. The gene expression data for individual replicates were averaged, normalized to zero-mean and unit variance, and subjected to clustering into six clusters, using Pearson correlation as the distance metric and 500 as the maximum number of iterations.

### RT-qPCR analysis

cDNA was synthesized with the TranScriba cDNA synthesis kit (A&A Biotechnology, Poland) using 1.5 μg of total RNA per reaction and random hexamer primers. RT-qPCR reactions were performed in a Roche LightCycler 480 using Hot FIREPol EvaGreen qPCR Mix Plus (Solis Biodyne). The reactions were performed with 0.15–0.3 μl of cDNA in a total volume of 20 μl. Three technical replicates were used for each gene/primer combination. The primers used to amplify target and reference genes are listed in S1 Table. Changes in the gene expression were calculated using the Pfaffl method [54] with normalization to the reference gene *nadB* (*PA0761*). RT-qPCR data represent mean for 3 biological replicates and 3 technical replicates. All RT-qPCR experiments were repeated twice and representative experiments are shown.

### Western blotting

Cultures were grown on L broth to OD_600_ 0.4–0.6. Cells were harvested by centrifugation and stored at -20^°^C. For each sample a small aliquot was used to estimate the number of c.f.u. ml^-1^ by plating appropriate dilution of the culture on L agar plates. Pellets were resuspended in 10 mM Tris-HCl (pH 8.0), 1 M NaCl, 0.1 mM EDTA, 5% glycerol. For each sample different volume of the buffer was used to compensate the initial differences in c.f.u. ml^-1^. Samples were sonicated and aliquots of cleared extracts corresponding to 10^9^ cells were separated by SDS-PAGE on 12% polyacrylamide gels. Additionally, His_6_-ParB, purified as described [37], was loaded on each gel. Separated proteins were transferred onto nitrocellulose membranes. The blots were subjected to a two-step immunoreaction including application of rabbit polyclonal anti-ParB antibodies followed by incubation with goat anti-rabbit antibodies conjugated with alkaline phosphatase and were developed by addition of NBT/ BCIP mixture. Band intensity was estimated using ImageJ for at least 3 biological replicates.

### Biofilm formation assay

Biofilm formation in static cultures was assayed as described previously with minor modifications [55]. Overnight cultures of *P*. *aeruginosa* strains in three biological replicates were diluted 1:100 in L broth with or without 0.02% arabinose. 75 μg ml^-1^ chloramphenicol was added to maintain the plasmids. 100 μl aliquots of each diluted culture were transferred into 9 wells of a 96-well flat-bottom polystyrene microplate (Greiner Bio-One). Sterile L broth was used as a negative control. The plate was incubated without shaking at 37°C until OD_600_ reached approximately 0.5. The optical density OD_600_ was measured using a plate reader (BioTek Synergy HT) and bacterial cells, which did not adhere to the surface, were gently removed by aspiration with a pipette. Wells were washed twice with 100 μl of PBS (15 mM KCl, 150 mM NaCl, 10 mM NaPi pH 7.4) and biofilm in each well was stained with 100 μl of 0.1% crystal violet solution. After 15 min incubation at room temperature, crystal violet solution was aspirated and wells were rinsed three times with distilled water and twice with 100 μl of PBS. Subsequently, 100 μl of 96% ethanol was added to each well and left for 10 min to dissolve the crystal violet. Well content was mixed by pipetting and OD_590_ was measured using a plate reader and 96% ethanol as a blank. Biofilm formation is presented as proportion OD_590_/OD_600_.

### β-galactosidase activity assays

β-galactosidase activity was measured as described before [42]. Extracts were prepared from cells grown to OD_600_ 0.4–0.6 in L broth with 75 μg ml^-1^ chloramphenicol, with or without 0.1% arabinose.

## Results

### Impact of *parB* overexpression on gene expression in *P*. *aeruginosa*

**Fig 1:**
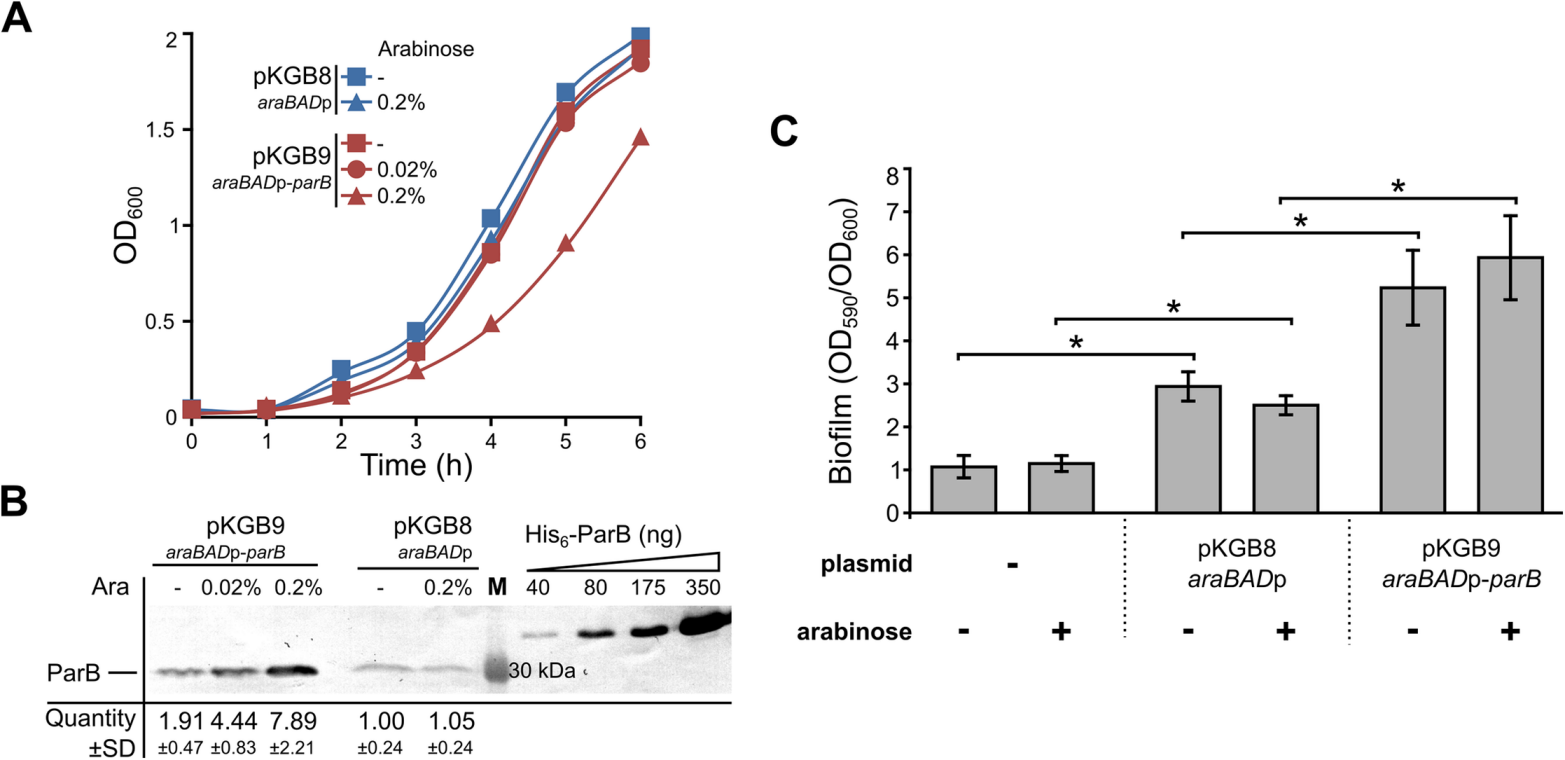
Effects of ParB excess in *P*. *aeruginosa*. (**A**) Growth of *P*. *aeruginosa* PAO1161 (pKGB8 *araBAD*p) and PAO1161 (pKGB9 *araBAD*p-*parB*) strains in L broth with different arabinose concentrations. Data represent mean OD_600_. (**B**) Western blot analysis of ParB levels in the tested strains. Each lane contains extract from 10^9^ cells. Blots were subjected to immunodetection using primary anti-ParB antibodies. Representative blot is shown. Signals on the blots were quantified. Data represent mean ParB level ±SD relatively to the control strain PAO1161 (pKGB8). Purified His_6_-ParB was used to generate standard curves. M–molecular weight marker. **(C**) Biofilm formation in the static cultures of PAO1161, PAO1161 (pKGB8) and PAO1161 (pKGB9). Strains were grown without or with 0.02% arabinose until OD_600_ 0.5. Biofilm was stained with crystal violet and assessed by measurement of OD_590_. Data represent mean OD_590_/OD_600_ ratio ±SD from 3 biological replicates. *—*p*-value < 0.05 in two-sided Student’s *t*-test assuming equal variance.

Our previous microarray analysis revealed altered expression of 1166 genes in ParB-deficient cells of *P*. *aeruginosa* relative to the wild type PAO1161 (WT) cells [39]. To complement this study, here we analysed the transcriptome of ParB -overproducing cells. To control the level of *parB* expression we constructed a pKGB8 expression vector based on the broad-host-range pBBR1MCS-1 [56], which contains the arabinose (Ara) inducible promoter *araBAD*p [49]. Analysis of the growth of PAO1161 (pKGB9 *araBAD*p-*parB*) and PAO1161 (pKGB8) cells in the presence of different concentrations of Ara revealed that induction by Ara ≤0.02% did not affect the growth (Fig 1A). A slight increase in the ParB level (≤2-fold) was observed even in non-induced cells of PAO1161 (pKGB9) relative to the cells containing the empty vector according to Western blot analysis (Fig 1B). Addition of 0.02% Ara to the medium resulted in a 5-fold increase in the ParB amount per cell in mid-log phase cultures of PAO1161 (pKGB9) in comparison to PAO1161 (pKGB8) (Fig 1B). Such ParB excess did not affect the frequency of anucleate cells formation (<0.1%), cell size, colony morphology or swimming and swarming motility (S1A–S1C Fig) but led to altered biofilm formation under specific conditions (Fig 1C, discussed below).

To determine the impact of an increased ParB level on the transcriptome a microarray analysis was performed on RNA isolated from PAO1161 (pKGB9 *araBAD*p-*parB*) cultures grown under selection in L broth with 0.02% Ara (higher ParB overproduction, hereafter referred to as ParB*+++*) or without Ara (mild ParB overproduction, hereafter called ParB*+*) as well as from PAO1161 (pKGB8) cells grown under selection in L broth with 0.02% Ara (empty vector control, hereafter EV) and PAO1161 cells grown in L broth without Ara (wild-type control, hereafter WT). A principal component analysis of the microarray data revealed that the biological replicates for WT and EV form 2 separate groups whereas the biological replicates of ParB*+* and ParB*+++* form together a separate group, distinct from WT and EV samples (S2 Fig).

**Fig 2:**
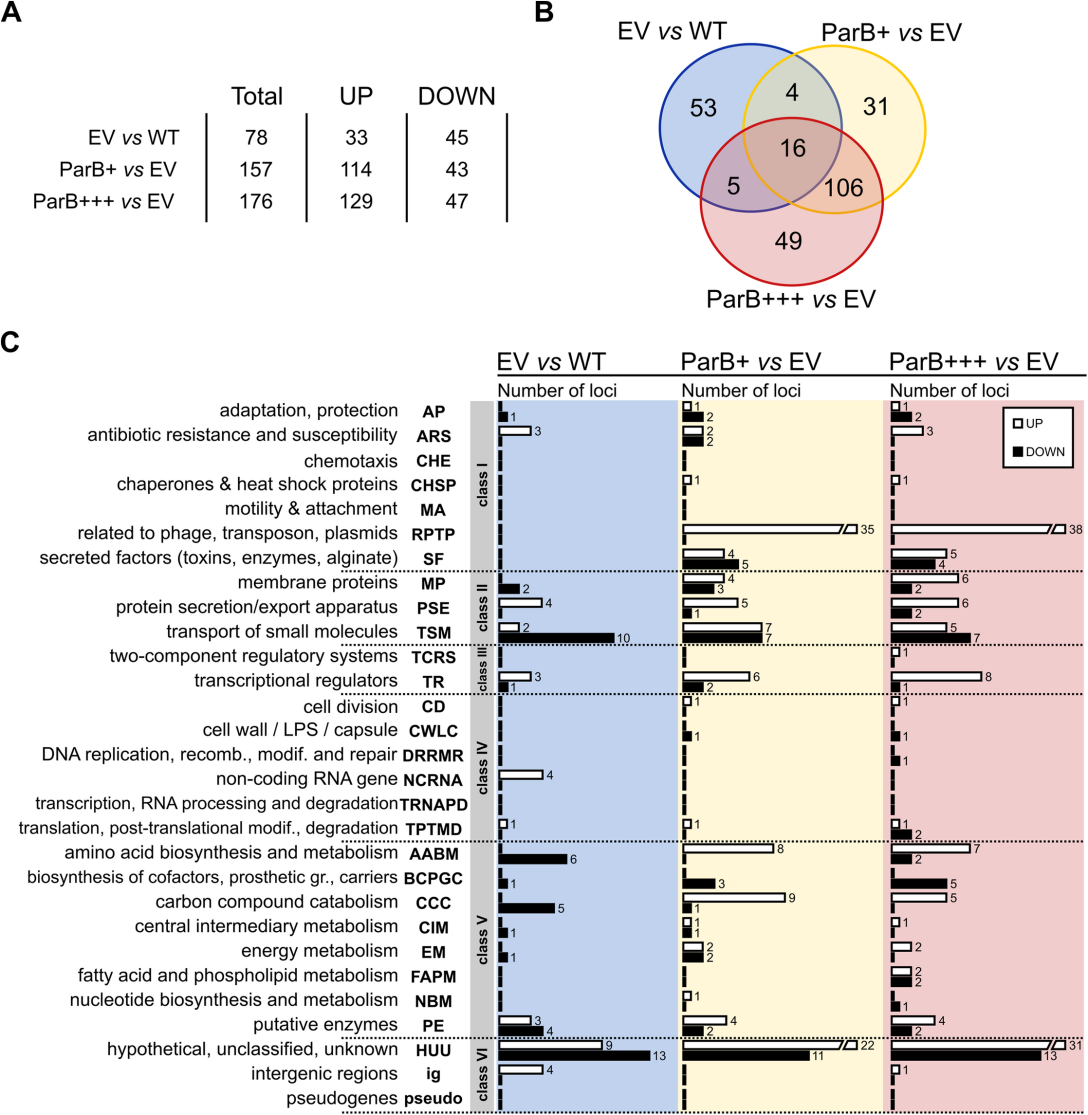
Transcriptome changes in response to ParB overproduction. (**A**) Statistics of loci with significant expression change (FC<-2 or >2, *p*-value <0.05). RNA was isolated from PAO1161 cultures grown in L broth without Ara (WT), PAO1161 (pKGB8 *araBAD*p) cultures grown under selection in L broth with 0.02% Ara (empty vector control, EV), PAO1161 (pKGB9 *araBAD*p-*parB*) cultures grown under selection in L broth without Ara (mild ParB excess, ParB+) or with 0.02% Ara (higher ParB excess, ParB*+++*). (**B**) Venn diagram for sets of loci with significant expression change between EV *vs* WT, ParB+ *vs* EV and ParB+++ *vs* EV. (**C**) Classification of loci with altered expression according to PseudoCAP categories [57]. When a gene was assigned to multiple categories, one category was arbitrarily selected (S2 Table). The PseudoCAP categories were grouped into six classes as marked. White and black bars correspond to the numbers of respectively, upregulated and downregulated genes in a particular category.

Comparative transcriptome analysis of EV *vs* WT revealed 70 genes, four tRNA genes, and four intergenic regions with an altered expression in response to the presence of the vector [fold change (FC) <-2 or >2, *p*-value <0.05] (Fig 2A, S2 Table). 175 genes and 1 intergenic region displayed statistically significant difference in expression between ParB*+++* and EV cells using the same criteria. 155 out of 175 genes displayed an altered expression only in the response to the ParB abundance since they were not identified in the EV *vs* WT analysis. The expression of the remaining 20 genes and one intergenic region was affected by the presence of vector as well as ParB overproduction in ParB+++ cells (Fig 2B, S2 Table). The comparative analysis of ParB*+* and EV transcriptomes revealed altered expression of 157 genes. There was a major overlap between the transcriptomic changes in ParB+ and ParB+++ cells as 122 genes showed similar responses in the ParB+ and ParB+++ cells (Fig 2B, S2 Table). This is not surprising as the transcription of *parB* (*PA5562*) itself was increased 4- and 12- fold in ParB*+* and ParB*+++* cells, respectively, in comparison to the EV cells.

Interestingly, there was also a set of 35 genes with expression changed only in ParB+ cells relative to EV cells but not in ParB+++. Closer inspection of the expression changes of these genes revealed three subgroups (S3A Fig, S2 Table): i/ 15 genes were also altered in ParB+++ transcriptome but with a slightly lower fold change (FC>1.6 or FC<-1.6, *p*-value <0.05) and were cut off from the common pool of ParB -affected genes using the criterion of FC, ii/ 6 genes despite their high FC were eliminated in ParB+++ vs EV analysis because of slightly higher variation of their expression in biological replicates (0.05< *p*-value <0.1), iii/ remaining 14 genes seemed to be not altered in ParB+++ transcriptome. Whereas ParB+++ and EV strains were grown under the same conditions (chloramphenicol and 0.02% arabinose), ParB+ cells were not exposed to arabinose. To estimate the impact of inducer on gene expression PAO1161 (pKGB8) was grown in medium with or without 0.02% Ara and RT-qPCR analysis of eight loci from the group of 35 genes in question, representing all three subgroups, was performed. The expression of only one gene, *PA2113* (from the bi-cistronic operon *PA2113-2114*) was altered in response to Ara presence in the medium (S3B Fig). *PA2113-2114* belong to the third subgroup in the expression pattern analysis (see above) but also to a distinct set of 4 genes common for EV *vs* WT and ParB+ *vs* EV lists (Fig 2B, S2 Table), hence it is likely that these four genes appeared in our analysis due to the influence of Ara on their expression. Presence of Ara had no influence on the expression of 7 out of 8 genes tested, which represent 14 genes due to the operon structures (S3B Fig), confirming that majority of genes with the expression altered in ParB+ cells relative to EV cells was identified due to their response to ParB excess.

The genes with altered expression were assigned to PseudoCAP categories [57] divided arbitrarily into six classes as described previously [39]. The comparison of the EV and WT transcriptomes revealed that the majority of affected genes belong to three groups: Class II comprising genes encoding membrane proteins, proteins involved in transport of small molecules, and protein secretion systems (18 genes), Class V comprising genes involved in metabolic pathways (21 genes), and Class VI with 22 HUU genes (hypothetical, unclassified, unknown) (Fig 2C, S2 Table). Similarly, most of the genes with altered expression in ParB overproducing cells (ParB+++ and ParB+) relative to EV cells were assigned to the same three classes (II, V and VI). However, almost one third of the genes with expression altered in response to ParB overproduction fall into Class I, mainly to PseudoCAP categories RPTP (related to phage, transposon, plasmids) and SF (secreted factors: toxins, enzymes, alginate). These data suggest that an increased ParB level leads to the activation of stress response.

### Different expression patterns of genes affected by ParB excess

**Fig 3:**
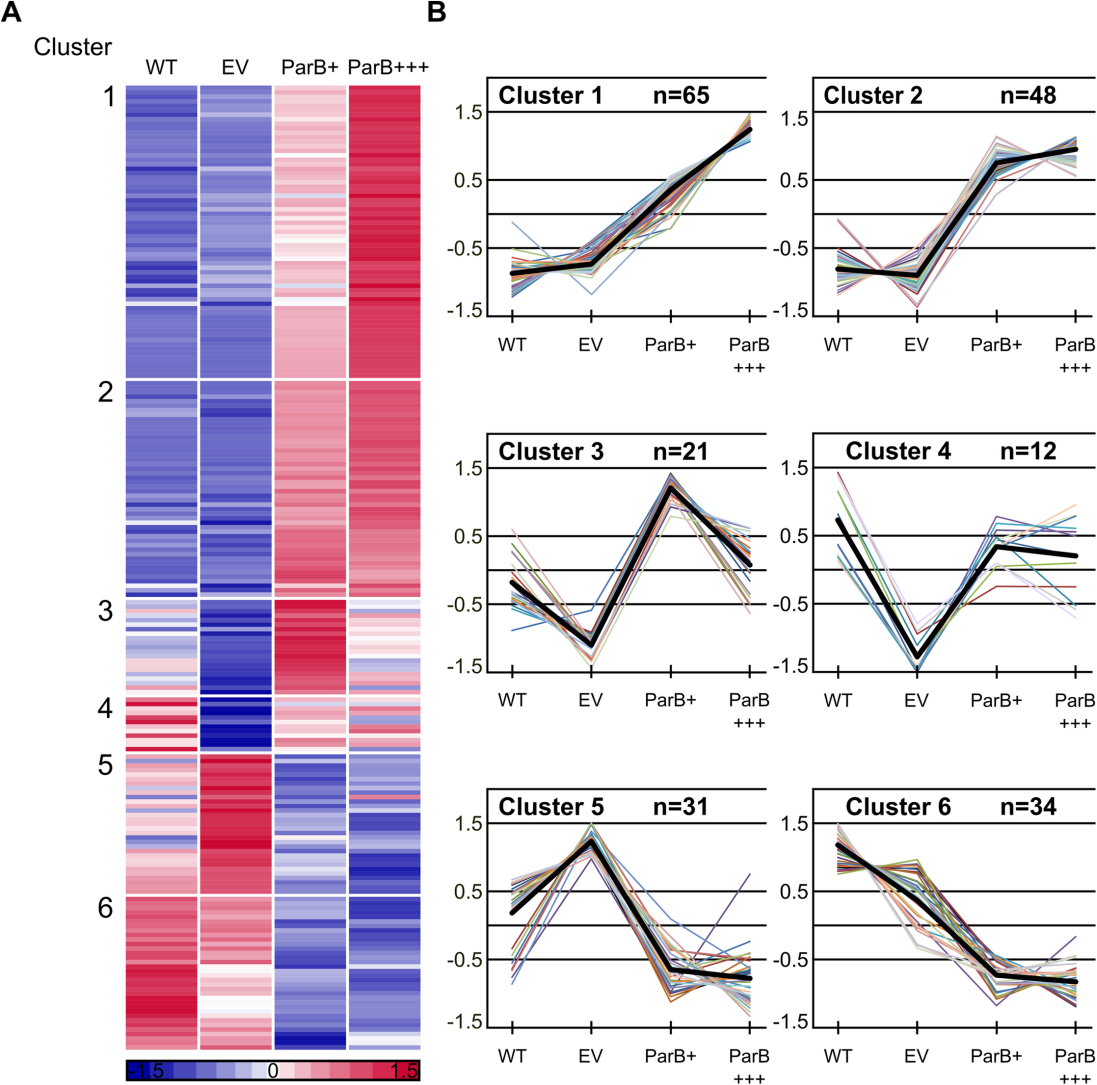
K-means clustering of microarray data. Expression data for replicates for each of 211 loci displaying altered expression in ParB overproducing cells were averaged, normalized to zero mean and unit variance and grouped into six clusters. (**A**) Expression profiles of individual genes grouped according to the results of K-means clustering. Each horizontal line represents one gene. Red and blue denote that the expression is respectively, above or below the mean expression of a gene across the data set. (**B**) Expression profiles for genes in each cluster. Y-axis represents the difference between expression of a particular gene in tested conditions and the mean expression of this gene in all 4 conditions presented as the number of standard deviations that a particular data point differs from the mean. Thick black lines represent the cluster centres. The genes from each cluster and their expression levels in different cells are listed in S3 Table.

To systematize the impact of the vector as well as the increased level of ParB on the PAO1161 transcriptome a K-means clustering analysis was performed [52]. The two lists of altered genes (ParB+ vs EV and ParB+++ vs EV) were combined yielding 211 unique loci (S2 Table). Expression data for replicates for each of these loci in the four strains/ growth conditions (WT, EV, ParB+, ParB+++) were averaged, normalized to zero mean and unit variance and grouped into six clusters based on the similarity of their expression patterns (Fig 3A and 3B).

Clusters 1 to 4 contain genes that are upregulated in ParB*+* and/or ParB+++ cells relative to EV cells and clusters 5 and 6 the downregulated ones. A detailed description of the genes in each cluster as well as their mean expression levels in the four conditions are given in S3 Table.

To validate the expression patterns obtained from the microarray analysis the transcript level for selected genes from each cluster in various samples was analysed using RT-qPCR. Importantly, for all tested 12 genes their relative levels of expression detected by RT-qPCR correlated nearly perfectly with the microarray data (Table 3).

**Table 3:** Validation of the microarray data by RT-qPCR.

		WT		EV	ParB+		ParB+++	
Cluster	Gene ID	MA	qPCR	qPCR	MA	qPCR	MA	qPCR
1	*PA0612*	0.66	0.76 ± 0.22	1 ± 0.06	6.54	5.62 ± 0.97	10.57	9.69 ± 3.3
1	*PA0985*	0.83	0.87 ± 0.16	1 ± 0.32	3.57	5.7 ± 1.67	5.02	7.1 ± 2.14
1	*PA4635*	0.60	0.48 ± 0.08	1 ± 0.12	2.95	1.42 ± 0.47	4.63	2.39 ± 1.12
1	*PA5562*	0.93	0.94 ± 0.21	1 ± 0.08	4.31	2.49 ± 0.43	12.06	17.3 ± 3.21
2	*PA2432*	0.62	0.57 ± 0.18	1 ± 0.29	8.36	5.13 ± 1.13	7.90	6.74 ± 0.1
2	*PA3661*	1.16	1.15 ± 0.45	1 ± 0.18	17.50	5.78 ± 2.23	23.34	8.39 ± 4.41
2	*PA5471*	0.24	0.28 ± 0.08	1 ± 0.25	2.24	2.01 ± 0.66	2.50	2.34 ± 0.34
3	*PA2008*	2.44	3.03 ± 1.06	1 ± 0.27	6.26	7.91 ± 1.36	3.81	3.91 ± 0.09
4	*PA4659*	1.73	0.97 ± 0.13	1 ± 0.13	1.83	2.23 ± 0.22	2.13	2.06 ± 0.1
5	*PA0996*	0.75	0.57 ± 0.01	1 ± 0.15	0.58	0.63 ± 0.22	0.31	0.33 ± 0.04
5	*PA1003*	0.87	0.73 ± 0.13	1 ± 0.24	0.72	0.58 ± 0.05	0.45	0.44 ± 0.04
6	*PA0011*	0.96	1.01 ± 0.2	1 ± 0.24	0.37	0.41 ± 0.03	0.20	0.24 ± 0.00

Relative transcript level of selected genes in replicates of *P*. *aeruginosa* cultures assessed by microarrays and RT-qPCR. WT—PAO1161 grown in L broth, EV—PAO1161 (pKGB8) grown in L broth with 0.02% Ara, ParB+—PAO1161 (pKGB9) grown in L broth and ParB+++—PAO1161 (pKGB9) grown in L broth with 0.02% Ara. Microarray (MA) data represent mean expression and RT-qPCR (qPCR) data represent mean ±SD from three biological replicates.

### Genes upregulated in response to *parB* overexpression

Clusters 1 (64 genes and one intergenic region), 2 (48 genes), 3 (21 genes) and 4 (12 genes) contain loci upregulated in response to ParB overproduction. For eight of these loci an increased transcript level is also observed in EV cells relative to WT cells (S3 Table, Table 4). Among these loci are *PA5471* gene encoding ArmZ, an inhibitor of MexZ which negatively regulates the *mexXY* operon encoding the multidrug efflux pump MexXY [58,59] and *pyeR* (*PA4354*) encoding a transcriptional regulator of biofilm formation [60]. Additionally, Cluster 1 contains the *arr* (*PA2818*) gene predicted to encode a phosphodiesterase whose substrate is cyclic di-guanosine monophosphate (c-di-GMP), a bacterial secondary messenger that regulates cell surface adhesiveness, virulence and biofilm formation [61].

**Table 4:** Selected genes whose expression was identified as ParB regulated.

ID		Gene	Fold change			Cluster	Description
			EV *vs* WT	ParB+ *vs* EV	ParB+++ *vs* EV		
**Genes induced by the presence of vector as well as ParB excess**							
*PA0805*			3,3		2,2	1	hypothetical protein
*PA1394*			2,1	2,0	2,7	1	hypothetical protein
*PA2288*			2,2	2,3	4,2	1	hypothetical protein
*PA4354*	*pyeR*		3,2		2,3	1	ArsR-family transcriptional repressor
*PA4826*			2,6		2,5	1	hypothetical protein
*PA5470*			7,1	2,3	3,0	1	probable peptide chain release factor
*PA5471*	*armZ*		4,1	2,2	2,5	2	MexZ anti-repressor
**Selected phage-related genes and pyocins**							
*PA0610*	*prtN*			2,8	5,4	1	transcriptional regulator PrtN
*PA0612*	*ptrB*			6,5	10,6	1	repressor PtrB
*PA0907*	*alpA*			3,4	4,3		transcriptional regulator
*PA0985*	*pyoS5*			3,6	5,0	1	pyocin S5
*PA1150*	*pys2*			2,9	4,1	1	pyocin S2
*PA3866*				2,6	4,1	1	Pyocin S4
**Genes related to type VI export**							
*PA0083*	*hsiB1*			2,0	2,3	2	sheath protein, Hcp secretion island I
*PA0084*	*tssC1*			2,2	2,1	2	TssC1, Hcp secretion island I
*PA0085*	*hcp1*			2,3	2,2	2	Hcp1, Type VI secretion system effector
*PA0087*	*tssE1*				2,2	2	component of Hcp secretion island I
*PA0089*	*tssG1*			2,2	2,5	2	component of Hcp secretion island I
*PA0090*	*clpV1*			2,7	2,7	2	putative ATPase, Hcp secretion island I
*PA0091*	*vgrG1a*			2,6	2,8	2	component of Hcp secretion island I
*PA0095*	*vgrG1b*			2,1		4	secreted protein of unknown function
*PA1844*	*tse1*				2,0	1	toxin
*PA1845*	*tsi1*			2,2	2,6	2	immune protein
**Genes regulated by BexR**							
*PA2432*	*bexR*			8,4	7,9	2	bistable expression regulator
*PA2433*				3,4	4,5	1	hypothetical protein
*PA1202*				6,9	5,8	2	probable hydrolase
*PA1203*				3,3	3,2	2	hypothetical protein
*PA1204*				2,3	2,2	2	NAD(P)H quinone oxidoreductase
*PA1205*				2,5	2,3	2	conserved hypothetical protein
*PA2698*				2,1	2,1	2	probable hydrolase
*PA1337*	*ansB*				-2,0	6	glutaminase-asparaginase
*PA1338*	*ggt*				-2,5	6	gamma-glutamyltranspeptidase precursor
*PA1340*	*aatM*				-2,8	6	putative amino acid transporter
*PA1341*	*aatQ*				-3,1	6	putative amino acid transporter
**Genes involved in PQS synthesis**							
*PA0996*	*pqsA*				-3,2	5	PqsA, probable coenzyme A ligase
*PA0997*	*pqsB*				-3,5	5	PqsB, probable beta-keto-acyl-acyl-carrier protein synthase
*PA0998*	*pqsC*			-2,0	-3,5	5	PqsC, probable beta-keto-acyl-acyl-carrier protein synthase
*PA0999*	*pqsD*			-2,1	-3,8	5	3-oxoacyl-[acyl-carrier-protein] synthase III
*PA1000*	*pqsE*			-2,4	-3,7	5	quinolone signal response protein
*PA1001*	*phnA*			-2,5	-4,6	5	anthranilate synthase component I
*PA1002*	*phnB*				-3,1	5	anthranilate synthase component II
*PA1003*	*mvfR*				-2,2	5	transcriptional regulator MvfR
**Pyochelin and pyoverdine biosynthesis genes**							
*PA4220*				-2,0		5	hypothetical protein
*PA4221*	*fptA*			-2,6		5	Fe(III)-pyochelin outer membrane receptor precursor
*PA4224*	*pchG*			-2,6	-2,5	6	pyochelin biosynthetic protein PchG
*PA4225*	*pchF*			-2,4	-2,9	6	pyochelin synthetase
*PA4228*	*pchD*			-2,9	-2,3	5	pyochelin biosynthesis protein PchD
*PA4229*	*pchC*			-2,2		5	pyochelin biosynthetic protein PchC
*PA4230*	*pchB*			-2,4	-2,1	6	salicylate biosynthesis protein PchB
*PA4231*	*pchA*			-2,5	-2,3	5	salicylate biosynthesis isochorismate synthase
*PA2384*				-3,0		6	transcriptional regulator
*PA2403*				-3,1		6	hypothetical protein
*PA2406*				-2,7	-3,2	6	hypothetical protein
*PA2409*				-3,0	-2,8	6	probable permease of ABC transporter
*PA2410*				-3,0	-3,2	6	hypothetical protein
*PA2411*				-2,2		5	probable thioesterase
**Transcriptional regulators**							
*PA0515*				3,1	3,0	4	probable transcriptional regulator
*PA1196*				3,7	3,5	2	probable transcriptional regulator
*PA2583*					2,3	1	sensor/response regulator hybrid
*PA4659*					2,1	4	probable transcriptional regulator
*PA4896*				-2,1		5	probable sigma-70 factor, ECF subfamily
**Genes adjacent to *parS1-4***							
*PA0004*	*gyrB*				-2,0	6	DNA gyrase subunit B
*PA0005*	*lptA*				-2,1	6	lysophosphatidic acid acyltransferase, LptA
*PA0006*					-2,1	6	conserved hypothetical protein
*PA0008*	*glyS*				-3,1	6	glycyl-tRNA synthetase beta chain
*PA0009*	*glyQ*				-2,5	6	glycyl-tRNA synthetase alpha chain
*PA0011*	*htrB1*			-2,7	-4,9	6	2-OH-lauroyltransferase
*PA0012*				-2,6	-3,7	6	hypothetical protein
*PA0013*				-3,4	-3,5	6	conserved hypothetical protein
**Genes adjacent to *parS6***							
*PA0492*				-6,7	-2,8	6	conserved hypothetical protein
*PA0493*				-4,3	-2,4	6	probable biotin-requiring enzyme
*PA0494*				-3,0	-2,2	6	probable acyl-CoA carboxylase subunit
*PA0495*				-3,6	-2,1	6	hypothetical protein

The biofilm formation was checked in the cultures of PAO1161 (pKGB9 *araBAD*p-*parB*) in comparison to PAO1161 (pKGB8) during growth under static conditions. No difference was observed between the strains in the overnight cultures (S1D Fig). Notably, a significant increase in biofilm formation was observed in dividing cultures of PAO1161 (pKGB9 *araBAD*p-*parB*) relative to PAO1161 (pKGB8), which correlates with the altered expression of genes involved in regulation of biofilm formation and identified in our study. No effect of arabinose presence on biofilm formation was detected (Fig 1C). Transcriptomic analysis also implicated that ParB excess could alter the response to antibiotic presence. The MICs for six antibiotics from β-lactam, aminoglycoside and fluorochinolone groups (piperacillin, ticarcillin, imipenem, gentamicin, tobramycin and ciprofloxacin) were tested but no significant differences between PAO1161 (pKGB9) and control PAO1161 (pKGB8) strains grown with and without arabinose were detected (S4 Table).

A remarkable feature of clusters 1 and 2, comprising the majority of ParB -upregulated genes (S3 Table), is a high proportion of phage-related genes (*PA0610*, *PA0612-0641*, *PA0643-0648*, *PA0717*, *PA0718*, *PA0907*, *PA0909-0911*) as well as pyocin-encoding genes (*PA0985*, *PA1150*, *PA3866*). Induction of bacteriophage genes and pyocin production in *P*. *aeruginosa* have been shown to be a hallmark of the SOS response [62], suggesting that *parB* overexpression induces a DNA damage signal. In this bacterium SOS response is coordinated not only by LexA (*PA3007*), but also by two structurally related repressors, PrtR (*PA0611*) and AlpR (*PA0906*) [62,63]. Significantly, expression of none of these genes is affected by ParB excess. PrtR is an inhibitor of *prtN* (*PA0610*), a transcriptional activator of pyocin synthesis genes [64]. *prtN* is induced 5.4-fold in ParB+++ cells suggesting a relief of the PrtR-mediated repression in these cells. Similarly, genes controlled by AlpR (*PA0907*, *PA0909-PA0911*) are induced in response to ParB overproduction. Further studies are required to determine the effect on the expression of the PrtR- and AlpR- regulated genes apparently without a change in the amounts of transcript level of these two repressors.

The ParB excess also induces genes encoding components of the Hcp secretion island HSI-1 [65] (*PA0083-0085*, *PA0087*, *PA0089-0091*) and the toxin/immunity proteins Tse1 (*PA1844)* and Tsi1 (*PA1845*). The HSI-1 together with two other secretion islands participate in *P*. *aeruginosa* virulence, inter- and intraspecies antagonism, biofilm formation, and stress sensing [66].

Overexpression of *parB* also results in an 8-fold increase in the expression of the transcriptional regulatory gene *PA2432* encoding a bistable response regulator BexR [67]. Six genes, *PA1202*, *PA1203*, *PA1204*, *PA1205*, *PA2433* and *PA2698*, previously identified as upregulated in response to BexR overproduction [67], are also upregulated in ParB- overproducing strains. Similarly, five genes downregulated in response to BexR overproduction, *PA0998*, *PA1337*, *PA1338*, *PA1340* and *PA1341*, are also downregulated in the analysed ParB -overproducing cells (Table 4).

**Fig 4:**
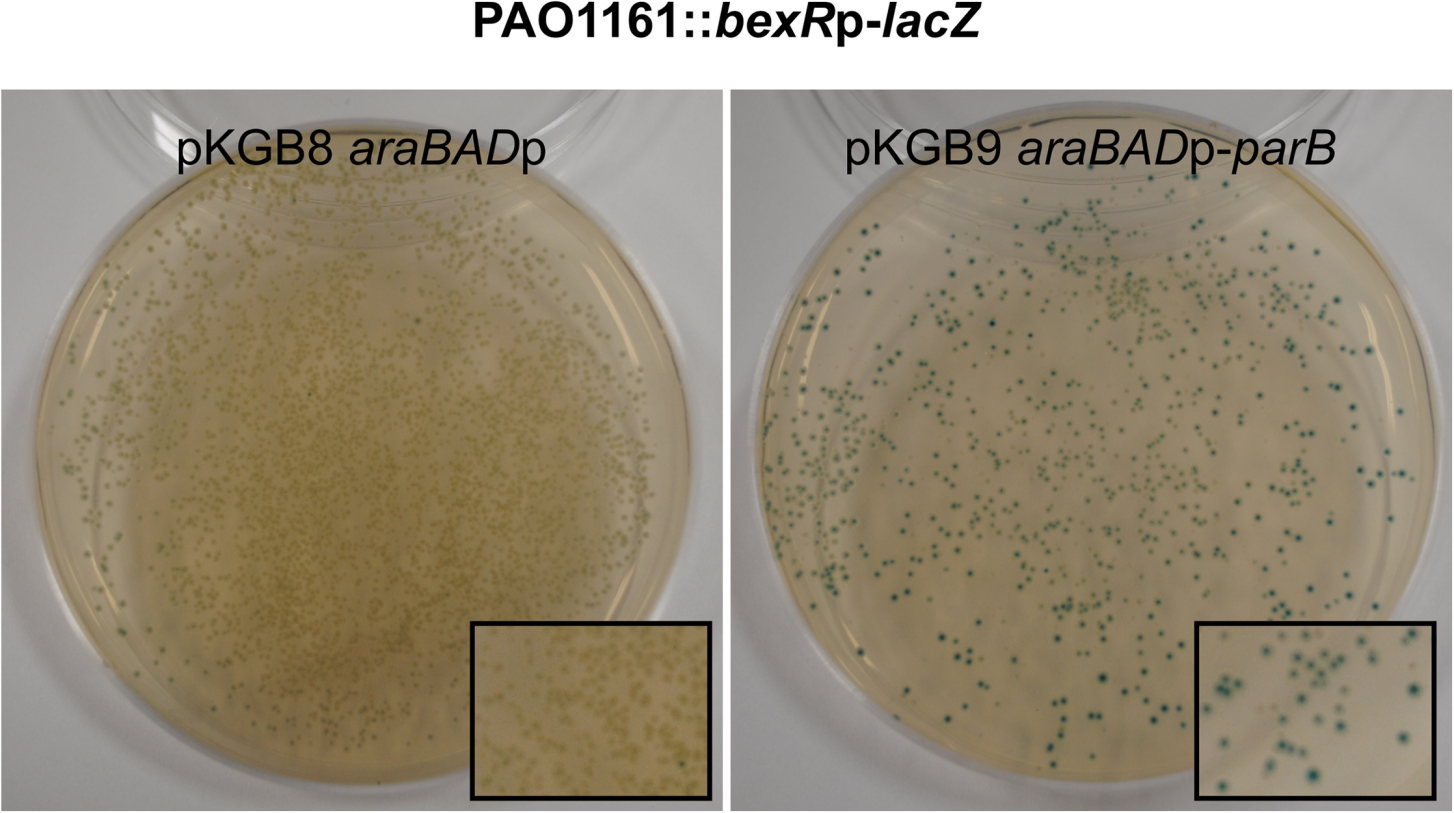
ParB excess induces the expression of chromosomal *bexR*p-*lacZ* transcriptional fusion. PAO1161::*bexR*p-*lacZ* strain contains *bexR*p-*lacZ* transcriptional fusion inserted in the intergenic region of PAO1161 genome. White colony from L agar with X-gal was inoculated and used as a recipient in conjugation with either S17-1 (pKGB8) or S17-1 (pKGB9 *araBAD*p-*parB*) donor cells. Conjugants were grown on selective L agar plates supplemented with X-gal. The photographs show representative plates of PAO1161::*bexR*p-*lacZ* with both plasmids.

The expression of *bexR* gene is known to be bistable, meaning that this gene switches between OFF and ON states in cells of a genetically identical bacterial population [67]. The microarray analysis was performed on three independent biological isolates of PAO1161 (pKGB9 *araBAD*p-*parB*) and all demonstrated a strong induction of *bexR* (*PA2432*) expression (*p*-value 3E-06) strongly suggesting that ParB excess modulates the BexR regulon through induction of *bexR* expression. To monitor the effect of ParB excess on promoter of *bexR* the strain PAO1161::*bexR*p-*lacZ* was constructed with the promoter-reporter cassette inserted in a non-coding region of the genome. A single colony (white on L agar with X-gal) was inoculated and the cells were used as the recipients in conjugation with either S17-1 (pKGB8) or S17-1 (pKGB9 *araBAD*p-*parB*). Conjugants were plated on selective medium containing X-gal to visualize the expression of *bexR*p-*lacZ* transcriptional fusion. Conjugants PAO1161::*bexR*p-*lacZ* (pKGB8) formed typical white/transparent colonies with a very low frequency of blue ones (less than 0.1%) whereas majority of PAO1161::*bexR*p-*lacZ* (pKGB9) conjugants formed blue colonies (Fig 4), confirming that ParB induces *bexR*p expression.

Clusters 3 and 4 group 33 genes with mRNA level reduced in response to plasmid/ chloramphenicol/ arabinose (EV) but then increased by ParB excess. Twelve of the 33 genes in this group belong to the PseudoCAP category carbon compounds catabolism (CCC) and eight genes to the amino acid biosynthesis and metabolism (AABM) category (S3 Table). The altered expression of genes from these categories suggests that ParB overproduction may interfere with primary metabolism as an adaptive measure. Interestingly, three out of four putative arabinose-dependent genes have also been classified into cluster 4. Additionally, cluster 4 contains two putative transcriptional regulators: *PA0515*, which is a part of the *nir* operon (denitrification operon), and *PA4659* with unknown function.

### Genes downregulated in response to *parB* overexpression

Clusters 5 (31 genes) and 6 (34 genes) contain genes downregulated in response to ParB overproduction but differ from each other by a moderate induction in EV cells seen in Cluster 5. This cluster contains all genes from two operons, *pqsABCDE* (*PA0996-1000*) and *phnAB* (*PA1001* and *PA1002*), involved in the production of *Pseudomonas* quinolone quorum sensing signal (PQS) [68,69]. Interestingly, the same cluster contains *PA1003* (*mvfR/pqsR*) which encodes a positive regulator of the both operons [70,71], suggesting that the downregulation of the *pqs* and *phn* operons is a direct result of lower *mvfR* expression. Interestingly, MvfR also acts as a negative regulator of type VI secretion system HSI-1 [66], components of which are upregulated in response to ParB excess (Table 4 and see above).

Five genes (*PA4220*, *PA4221*, *PA4228*, *PA4229*, *PA4231*) from Cluster 5 and three genes (*PA4224*, *PA4225*, *PA4230*) from Cluster 6 are involved in the biosynthesis and transport of a siderophore, pyochelin. Pyochelin synthesis in *P*. *aeruginosa* cells is positively regulated by the transcriptional regulator PA2384 [72], which is downregulated in ParB+ cells. Interestingly, biosynthesis of another siderophore, pyoverdine, also seems to be negatively affected by ParB excess as the genes *PA2403*, *PA2406*, *PA2409* and *PA2410* (Cluster 6) from the pyoverdine biosynthesis operon are significantly downregulated in ParB-overproducing cells (Table 4).

### Analysis of ParB-related transcriptional silencing around *parS* sites

**Fig 5:**
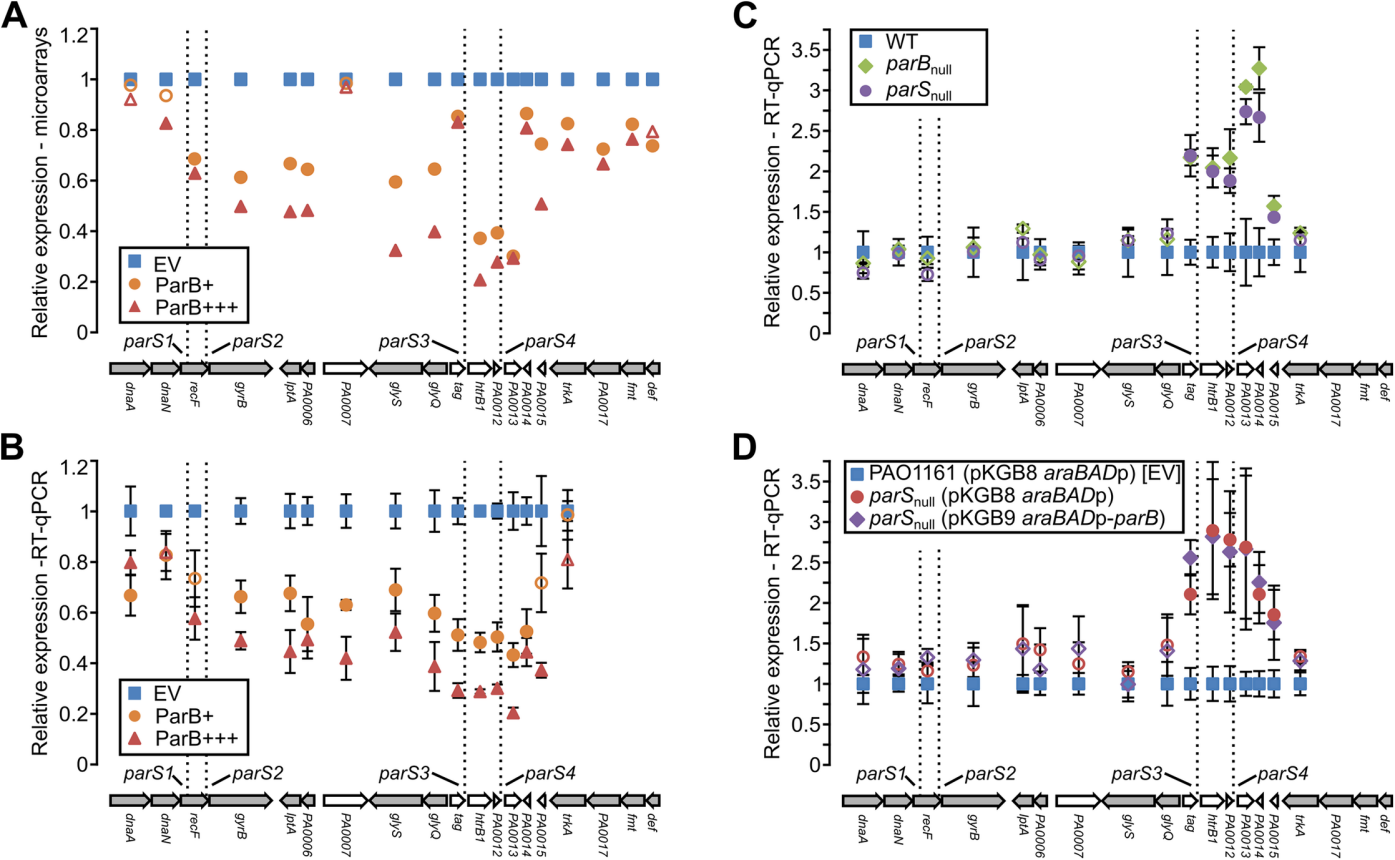
Influence of ParB on gene expression in the *parS1-4* region. (**A**) Mean level of expression of *dnaA*-*def* (*PA0001*-*PA0019*) genes in ParB+ and ParB+++ cells relative to EV as revealed by microarray analysis. Filled markers indicate statistically different expression relative to EV (*p*-value < 0.05 in ANOVA test). Arrangement of the genes in the chromosome is shown below. Operons (according to the DOOR 2.0 database [73]) are marked in grey. (**B**) Expression of *PA0001*-*PA0016* **(***dnaA*–*trkA*) genes in ParB+ and ParB+++ cells relative to EV cells. (**C**) RT-qPCR analysis of expression of *dnaA*–*trkA* genes in *parB*_null_ and *parS*_null_ strains relative to WT cells. Cells were grown in L broth. (**D**) RT-qPCR analysis of expression of *dnaA*–*trkA* genes in *parS*_null_ (pKGB9 *araBADp*-*parB*) and *parS*_null_ (pKGB8 *araBAD*p) relative to EV [PAO1161 (pKGB8 *araBAD*p)]. Cells were grown in L broth supplemented with chloramphenicol and 0.02% arabinose. RT-qPCR data represent mean ±SD from three biological replicates. Filled symbols indicate significantly different expression (*p*-value <0.05 in two-sided Student’s *t*-test assuming equal variance) relative to the control cells labelled as blue squares. The differences in expression of genes in *parS*_null_ (pKGB9) strain relative to *parS*_null_ (pKGB8) strain are not statistically significant.

Analysis of the microarray data revealed that ParB overproduction reduces the expression of a number of genes in close proximity of *parS1-4* (Table 4). Closer inspection of the expression changes in this region revealed that genes *PA0003-PA0015*, with the exception of *PA0007*, *tag* (*PA0010*) and *PA0014*, show a significant (30%-80%, *p*-value <0.05), ParB dose-dependent downregulation (Fig 5A). RT-qPCR analysis revealed that in fact all genes from *PA0004* to *PA0014* are subject to significant downregulation (Fig 5B), suggesting that ParB bound to *parS1*-*4* negatively influences the expression of adjacent genes. To verify this hypothesis we analysed the expression of these genes in the *parB*_null_ mutant, which does not produce ParB [18], and in the *parS*_null_ mutant in which the *parS1-4* sequences had been mutated, so the ParB binding to these sequences was impaired [38]. In the both mutants only genes from *PA0010* to *PA0015* displayed significantly increased expression (Fig 5C) indicating that at its native level ParB acts as a major negative regulator of genes adjacent to intergenic *parS3* and *parS4* but not for the genes adjacent to intragenic *parS1* and *parS2*. Regulation of the expression of genes in *parS1-parS4* region seems to be a direct consequence of ParB binding to *parS1*-*parS4* as ParB overproduction in *parS*_null_ cells did not lower the expression of *dnaA*–*trkA* (*PA0001*-*PA0016*) genes relatively to the control *parS*_null_ cells carrying empty vector (Fig 5D, also see below).

**Fig 6:**
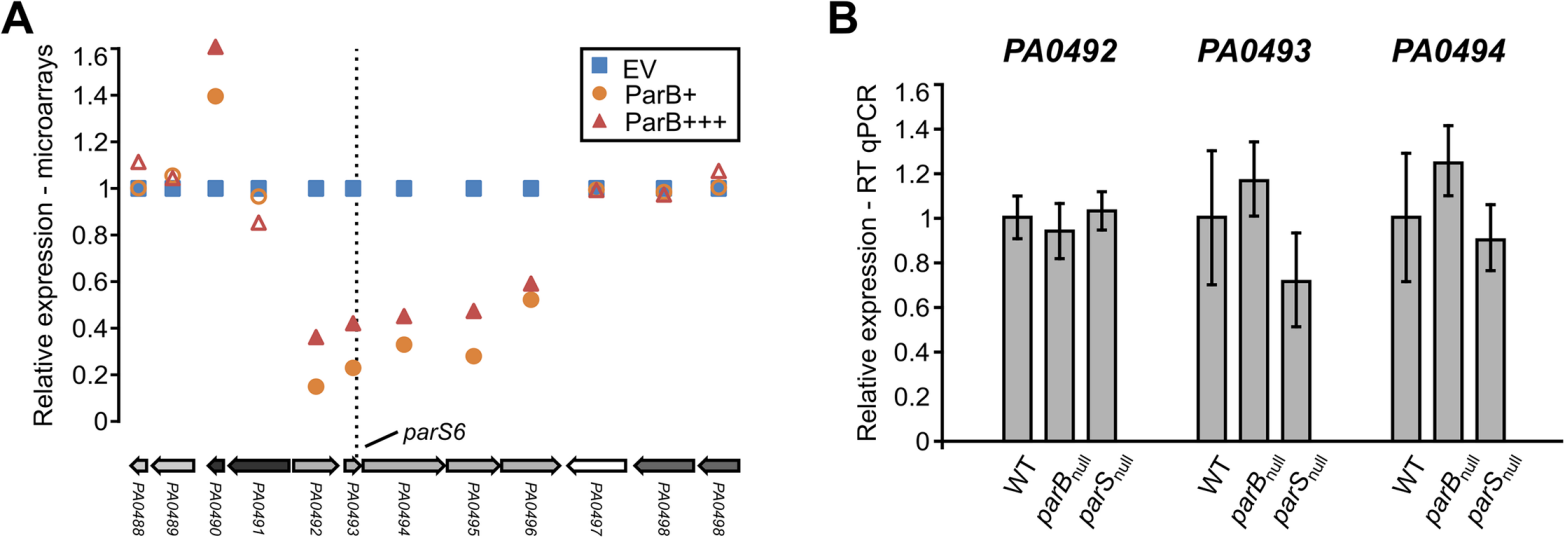
Influence of ParB level on expression of genes adjacent to *parS6*. (**A**) Mean level of expression of *PA0488*–*PA0498* genes in ParB+ and ParB+++ cells relative to EV cells (microarray data). Filled markers indicate statistically different expression relative to EV (*p*-value < 0.05 in ANOVA test). Operons (according to the DOOR 2.0 database [73]) are labelled with different shades of grey. (**B**) RT-qPCR analysis of expression of *PA0492*, *PA0493* and *PA0494* genes in *parB*_null_ and *parS*_null_ strains relative to WT cells. Data represent mean ±SD from three biological replicates. The differences between strains / conditions are not statistically significant (*p*-value > 0.05 in two-sided Student’s *t*-test assuming equal variance).

A similar inspection of the expression changes in proximity (±10 adjacent genes) of the remaining six *parS* sequences [38] revealed that overproduction of ParB lowers the expression of genes adjacent to *parS6* (Fig 6A, *PA0492-PA0496*) but not to the other *parS* sequences (S5 Table). However, the expression of *PA0492*, *PA0493* and *PA0494* as assayed by RT-qPCR was not significantly changed in *parB*_null_ or *parS*_null_ cells in comparison with the WT strain (Fig 6B), indicating that at its native abundance ParB is not a major effector of genes in this region.

### Regulation of *PA0011* and *PA0013* promoters by ParB interactions with *parS3* and *parS4* sequences

**Fig 7:**
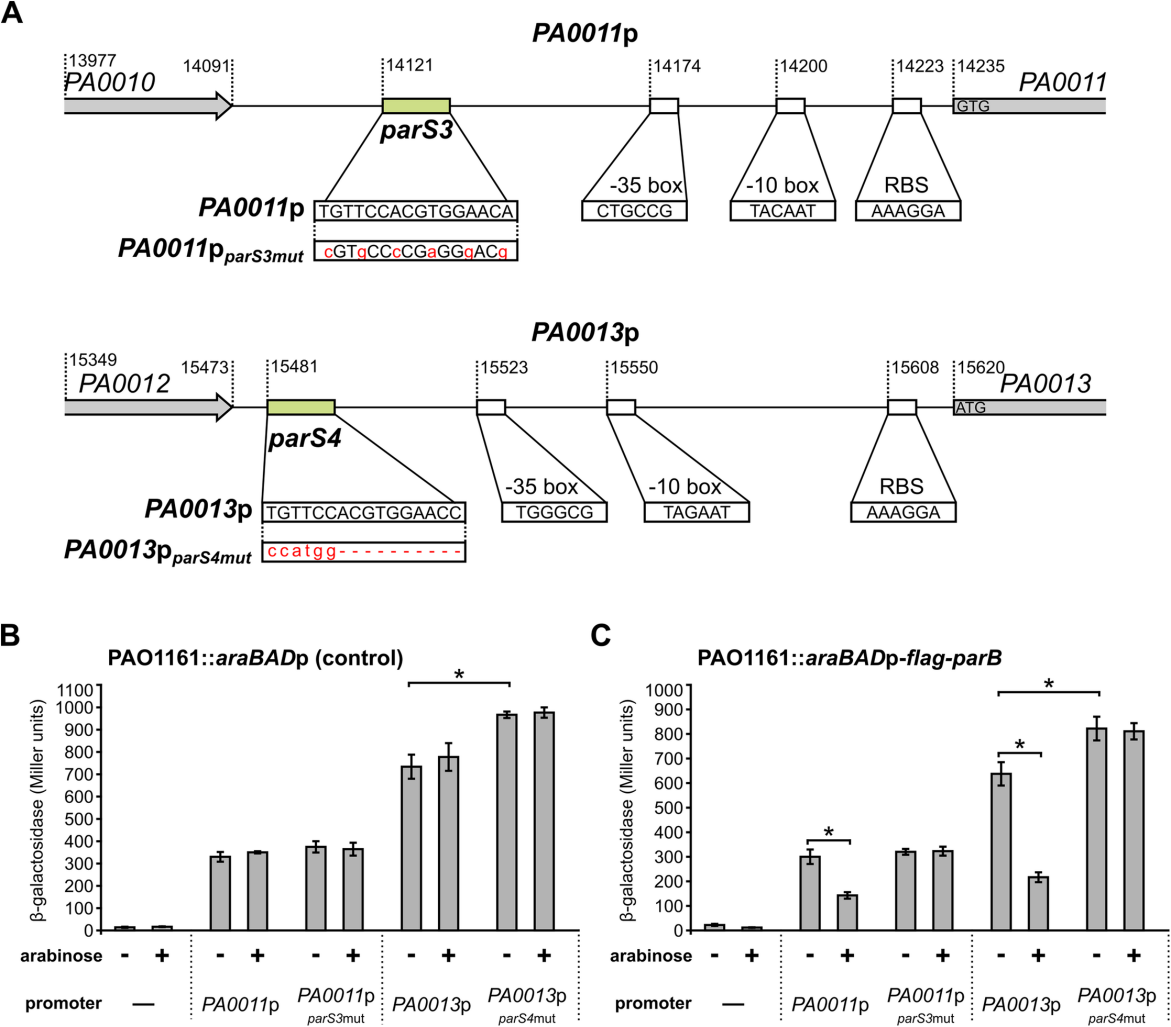
Influence of ParB on the activities of *PA0011* and *PA0013* promoters. (**A**) DNA sequences preceding *PA0011* (*PA0011*p) and *PA0013* (*PA0013*p) and their mutated versions, *PA0011*p_*parS3*mut_ and *PA0013*p_*parS4*mut_, cloned upstream of promoter-less *lacZ* cassette in pPJB132 are presented. Putative promoters’ motifs, RBS sequences and start codons are indicated. Promoter -35 and -10 boxes were predicted using BPROM [74]. β-galactosidase activity was measured in extracts from PAO1161::*araBAD*p (control strain) (**B**) and PAO1161::*araBAD*p-*flag*-*parB* (ParB-overproducing strain) (**C**) cells carrying pPJB132 derivatives as indicated. Data represent mean activity from at least three cultures ±SD. *—*p*-value < 0.05 in two-sided Student’s *t*-test assuming equal variance.

To further confirm that ParB binding to *parS3* and *parS4* affects the expression of adjacent genes the intergenic regions preceding *PA0011* and *PA0013* and their respective variants carrying mutated *parS3* and *parS4* (Fig 7A) [38] were cloned upstream of a promoter-less *lacZ* cassette in pPJB132, a derivative of pCM132 [50]. To produce an excess of ParB from the chromosome strain PAO1161::*araBAD*p-*flag-parB* was constructed with the expression cassette inserted in a non-coding region of the genome (Table 1). Growth of the PAO1161::*araBAD*p-*flag-parB* cells in a medium containing 0.1% arabinose results in the overproduction of Flag-ParB to a level that is not toxic for the cells (S4 Fig). All four pPJB132 derivatives were introduced into PAO1161::*araBAD*p-*flag-parB* as well as into control strain PAO1161::*araBAD*p with the empty expression cassette inserted in the same genomic position.

Analysis of the β-galactosidase activity in the transformants of the control strain revealed no effect of 0.1% arabinose on the promoters tested (Fig 7B). The nucleotide substitutions in *parS3* modifications (Fig 7A) did not affect the *PA0011*p activity (Fig 7B). Surprisingly, replacing *parS4* by a NcoI restriction site (deletion of 10 bp) resulted in a minor increase of the promoter strength in the *PA0013*p_*parS4*mut_*-lacZ* fusion (Fig 7B). DNA sequence analysis of promoters’ regions does not give a clear explanation of such effect (Fig 7A). The β-galactosidase activities detected in transformants of PAO1161::*araBAD*p-*flag*-*parB* were similar to those in the corresponding control strain and when the strains were grown without arabinose. However, upon induction of *flag*-*parB* expression with 0.1% arabinose a reduction of β-galactosidase activity was observed in PAO1161::*araBAD*p-*flag*-*parB* transformants carrying plasmids with the *PA0011*p-*lacZ* and *PA0013*p-*lacZ* transcriptional fusions but not plasmids with the *PA0011*p_*parS3*mut_-*lacZ* or *PA0013*p_*parS4*mut_-*lacZ* transcriptional fusions (Fig 7C). These data confirmed that ParB negatively regulates expression of these two promoters *in vivo* through interactions with *parS* sites.

## Discussion

In *P*. *aeruginosa*, partitioning protein ParB plays a non-essential but important role in segregation of chromosomes. Its binding to at least one of the four *parS* sequences in the *parS1-4* cluster, closest to *oriC*, is necessary and sufficient for accurate segregation [33,38]. The role of the remaining six *parS* sequences (four in the *ori* domain and two, *parS7* and *parS8*, close to the terminus domain [38]) is not fully understood. Ten similarly distributed *parS* sequences in *B*. *subtilis* have been shown to participate in SMC recruitment, condensation and juxtapositioning of chromosome arms [75]. Several reports have indicated that partitioning proteins may also modulate transcription of certain genes [35,36] but *P*. *aeruginosa* seems to be unique, as inactivation of *parA* or *parB* leads to large-scale changes of its transcriptome [39]. A lack or an excess of ParB protein is manifested by similar phenotypic changes, including erratic chromosome segregation, increased rate of formation of anucleate cells, cell elongation, disturbed division and defects in swarming and swimming [17,18,37]. To find out how such multiple effects arise, a microarray-based transcriptomic analysis was performed of PAO1161 derivatives in which ParB level was increased either 2-or 5- fold in comparison with the strain carrying empty vector. Since after binding to a specific *parS* sequence ParB has the ability to spread on the adjacent DNA, we mainly focused on expression of genes in the vicinity of *parS* sequences.

At tested ParB concentrations its binding to *parS* sites has no pronounced effect on expression of genes around them with the exception of genes located in proximity of *parS1-4* and *parS6*. Microarray and RT-qPCR data show convincingly that all the genes adjacent to *parS1-4* (*PA0003* to *PA0015*) are downregulated in the presence of ParB excess (Fig 3A and 3B). The interference of ParB with the expression of a vital replication operon (*dnaA-dnaN-recF-gyrB*), likely caused by ParB binding to *parS1*/*parS2* sites within the *recF* gene, may underlie the defects in cell division caused by a ParB excess larger than studied here. In contrast, in cells lacking a functional ParB protein or carrying mutated *parS1-4* sequences unable to bind ParB, RT-qPCR analysis revealed only upregulation of *PA0010*-*PA0015* genes, located in proximity of the intergenic *parS3* and *parS4*. Linking the regions preceding *PA0011* and *PA0013* to the promoter-less *lacZ* cassette confirmed the presence of functional promoters in the cloned fragments. Both promoters were repressed by ParB excess provided functional *parS3* and *parS4* were present, which confirmed that ParB binding directly affects transcription in this region.

The product of *PA0011*, the 2-OH-lauroyltransferase HtrB1 is involved in lipid A biosynthesis important for integrity of the outer membrane and cell envelope [76–78]. *htrB1* expression is induced by sub-inhibitory concentrations of different antibiotics [78] or growth at low temperature [77]. Inactivation of *htrB1* leads to pleiotropic effects, manifested by increased susceptibility to surfactants and antibiotics, impaired swarming motility, growth defects and induction of type III secretion system. Some of these effects are also observed under ParB excess. Conversely, expression of another HtrB homolog (*PA3242*) is significantly reduced in *parB*_null_ mutant [39]. Since inactivation of either of the *htrB* genes in *P*. *aeruginosa* PAO1 leads to motility defects [77,79], this could explain the impaired motility seen both when ParB is missing and when it is present in excess. Notably, also other genes adjacent to *parS3* and *parS4* encode proteins with putative roles in response to cellular stress. *tag* (*PA0010*) encodes a putative 3-methyladenine glycosidase I [80]. *PA0012* encodes a protein with a predicted PilZ domain, which could bind cyclic di-GMP, an important signalling molecule in *P*. *aeruginosa* [81,82]. Further studies are needed to characterize the biological functions of all the genes adjacent to *parS3* and *parS4* and the role of ParB in their regulation.

Apart from the genes in proximity to *parS3/*-*parS4*, also those close to *parS6* showed a reduced expression in response to ParB excess, however, their expression was not altered in *parB*_null_ and *parS*_null_ strains, suggesting that here the ParB involvement is a part of a more complex regulatory circuit. No changes were observed in the transcription of genes adjacent to *parS5* and *parS7*-*parS10* upon ParB excess. Indeed, a recent ChIP-seq analysis did not indicate binding of ParB to these sequences *in vivo* in cells grown in minimal medium [33], but instead showed the presence of nine additional ParB-bound sites. However, none of the genes adjacent to those regions changed expression in response to ParB excess in cells grown in conditions tested here (S5 Table). It is likely that the interaction of ParB with such secondary ParB binding sites, which is not required for chromosome segregation, may strongly depend on experimental conditions. It would be therefore interesting to compare ParB ChIP data obtained for cells grown in minimal vs. rich medium.

A comparison of the transcriptional changes in *parB -*deficient cells and cells expressing ParB excess identified 87 genes found in both studies (S6 Table) [39]. Expression of 83 genes similarly responded to either lack or abundance of ParB. Majority of them seem to be involved in the stress adaptation. Only *PA0293*, *PA1894*, *PA2113* and *PA2792* showed opposite trends of the response. These four genes are downregulated in *parB -*deficient cells and upregulated in response to ParB excess, hence it is unlikely that, akin to genes in the proximity of *parS3* and *parS4*, ParB directly affects their expression.

Our data indicate that ParB excess downregulates two operons *pqsABCDE* and *phnAB* involved in quinolone quorum sensing signalling [68,69], probably indirectly by repressing *PA1003* (*mvfR/pqsR*) coding for a transcriptional activator of the both operons [70,71]. Additionally, the ParB excess interferes with expression of key virulence determinants, two pathways involved in the production of the siderophores pyochelin and pyoveridine.

The extreme adaptability and excellent survival of *Pseudomonas* species have been claimed to originate from the plasticity of gene expression facilitated by the high number of transcriptional regulators encoded in their genomes [83,84]. ParB excess leads to the induction of several genes encoding known and putative transcriptional regulators (Table 4). Among them is *PA2432* encoding the bistable response regulator BexR [67]. Bistability refers to a phenotypic heterogeneity within an isogenic population which allows a fraction of cells to survive in otherwise lethal conditions [85]. The BexR regulon comprises a diverse set of up- and down- regulated genes related to virulence and quorum sensing [67]. Importantly, the *bexR* induction in response to ParB excess is highly reproducible and approaches the ~10-fold induction maximum found in the subset of cells which have switched on the BexR regulon [67]. The mobilization of pKGB9 (*araBAD*p-*parB*) and pKGB8 (*araBAD*p) plasmids into the test strain PAO1161::*bexR*p-*lacZ* with “OFF” phenotype demonstrated “ON” state of *bexR*p in nearly all conjugants with pKGB9 and in a tiny fraction of conjugants with pKGB8 (<0.1%). This confirms that the ParB excess induces the *bexR* gene in all cells rather than in a specific subpopulation only. So far the mechanism of *bexR* induction in response to ParB excess remains unknown.

Our study also revealed that ParB excess leads to the induction of bacteriophage and pyocin encoding genes, which in *P*. *aeruginosa* are hallmarks of the SOS response [62,86]. Whereas the SOS response had initially been linked exclusively to DNA damage signals, it was later established to be part of a broad stress reaction [87,88]. SOS response is tightly connected to cell growth inhibition, cell-cycle checkpoints and even programmed cell death [89–91]. The phage- and pyocins-related genes are among the genes responding similarly to the *parB* deficiency and ParB excess (S6 Table), suggesting that any imbalance in the ParA/ParB/*parS* system may trigger SOS response.

Altogether, our data indicate that the level of partitioning protein ParB in *P*. *aeruginosa* influences the expression of numerous genes including those involved in virulence, stress response and quorum sensing. Further studies are needed to decipher whether these effects are a direct result of ParB binding to DNA (specific or non-specific), interactions with other proteins or are caused indirectly, for example by topological changes.

## Supporting information

S1 Fig Phenotypic analysis of ParB overproducing strain PAO1161 (pKGB9 araBADp-parB) grown without and with 0.02% arabinose. **(A)** Overnight cultures of PAO1161 (pKGB9) and PAO1161 (pKGB8 *araBAD*p) were 100-fold diluted into L broth without and with 0.02% arabinose and grown to OD_600_ 0.6. Cells were collected for microscopic observations, fixed and stained with DAPI (4,6-diamidino-2-phenylindole) as described previously^1^. Cells were observed using fluorescent microscope Carl Zeiss Axio Imager.M2 with appropriate lens EC Plan-Neofluar 100x/1.30 Oil Ph 3 M27 and camera AxioCamMR5. Collected pictures were analyzed with the program AxioVision Rel.4.8.2 (Carl Zeiss). The average cell length (±SD) in [μm] were calculated for at least 500 cells. **(B)** Colony morphology of PAO1161 (pKGB9) and PAO1161 (pKGB8) strains was observed using stereomicroscope Nikon SMZ1500 after 24 h incubation at 37^°^C on L agar plates with 75 μg ml^-1^ chloramphenicol with 0.02% arabinose or without. Images were captured with NIS-Elements 2.10 software. **(C)** Motility of PAO1161 (pKGB9) and PAO1161 (pKGB8) strains was analyzed as previously described^1^. Strains were grown overnight under selection and without or with 0.02% arabinose. All sets of plates for swimming and swarming tests were standardized by using the same volume of medium. The plates were inoculated with a sterile toothpick incubated for 48 h at 30^°^C and photographed. In the case of swimming plates, diameters of the swimming zones are indicated. **(D)** Biofilm formation in the overnight static cultures of PAO1161, PAO1161 (pKGB8) and PAO1161 (pKGB9). Strains were grown without or with 0.02% arabinose. Biofilm was stained with crystal violet and assessed by measurement of OD_590_. Data represent mean OD_590_/OD_600_ ratio ±SD from 3 biological replicates. *—p-value < 0.05 in two-sided Student’s t-test assuming equal variance.^1^ Lasocki et al., J Bacteriol. 2007;189: 5762–5772. 10.1128/JB.00371-07
(TIF)

S2 Fig Principle component analysis (PCA) of data obtained from microarray analysis. (TIF)

S3 Fig Analysis of genes identified as altered in ParB+ *vs* EV but not ParB+++ *vs* EV. Abbreviation ParB+ corresponds to PAO1161 (pKGB9 *araBAD*p-*parB*) strain grown without arabinose, EV corresponds to PAO1161 (pKGB8 *araBAD*p) grown with arabinose and ParB+++ for PAO1161 (pKGB9 *araBAD*p-*parB*) cells grown with arabinose. (**A**) Fold change- and *p*- values for the 35 genes in ParB+ vs EV and ParB+++ vs EV analysis. First subgroup of genes with -1.6> FC >-2 or 1.6< FC <2 and *p*-value <0.05 in ParB+++ vs EV comparison is indicated in green. The second subgroup of genes with 0.05< *p*-value <0.1 in ParB+++ vs EV comparison is indicated in blue. The third subgroup of genes with *p*-value >0.1 and -1.5< FC < 1.5 is left uncoloured. Genes in operons are marked with arrows. (**B**) Impact of arabinose on the expression of selected genes. PAO1161 (pKGB8 *araBAD*p) cells were grown in L broth containing chloramphenicol with or without 0.02% arabinose. RT-qPCR was performed cDNA synthesized on RNA isolated from cells harvested at OD_600_ 0.5. Data represent mean ±SD from three biological replicates. Expression values for all genes are shown relative to the cells from cultures without arabinose. *—*p*-value < 0.05 in two-sided Student’s *t*-test assuming equal variance.
(TIF)

S4 Fig Analysis of PAO1161:: *araBAD*p-*flag*-*parB* strain. **(A)** Growth of *P*. *aeruginosa* PAO1161::*araBAD*p, PAO1161::*araBAD*p-*flag*-*parB* and PAO1161 strains in L broth with different arabinose concentrations. Data for PAO1161::*araBAD*p-*flag*-*parB* grown on 0.1% arabinose represent mean OD_600_ for three biological replicates ±SD. (**B**) Impact of 0.1% arabinose on the expression of *parB* in PAO1161::*araBAD*p-*flag*-*parB* relatively to PAO1161::*araBAD*p cells. Strains were grown in L broth with 0.1% arabinose. RT-qPCR was performed on RNA isolated from cultures harvested at OD_600_ 0.5. Data represent mean ±SD from three biological replicates. *—*p*-value < 0.05 in two-sided Student’s *t*-test assuming equal variance.
(TIF)

S1 Table Oligonucleotides used in this work. (DOCX)

S2 Table Transcriptomic changes between analyzed PAO1161 strains. (**A)** Comparison of PAO1161 (pKGB8 *araBAD*p) (EV) *vs* PAO1161 (WT). (**B)** Comparison of PAO1161 (pKGB9 *araBAD*p-*parB*) grown without arabinose (ParB+) *vs* EV. (**C)** Comparison of PAO1161(pKGB9 *araBAD*p-*parB*) grown with 0.02% arabinose (ParB+++) *vs* EV. (**D**) List of 211 loci displaying altered expression in ParB overproducing cells.
(XLSX)

S3 Table K-means clustering of ParB dependent loci. Mean levels of transcripts for all loci are shown relative to PAO1161 (pKGB8 *araBAD*p).
(XLSX)

S4 Table Influence of ParB overproduction on the antibiotic resistance of PAO1161. Upper panel demonstrates the growth test of PAO1161 (pKGB8) and PAO1161 (pKGB9) in the presence of a range of antibiotic concentrations (2-fold dilution steps) relatively to the standard *Pa* strain ATCC 27853. The bottom panel demonstrates the range of concentrations tested for chosen antibiotics and EUCAST 2016 (European Committee on Antimicrobial Susceptibility) breakpoint table for interpretation of MICs. Cells were grown in Mueller Hinton cation adjusted broth (Difco) supplemented with antibiotics and arabinose (Ara) as indicated. Chloramphenicol (Cm) at concentration of 75 µg ml^-1^ was added to maintain the plasmids.
(DOCX)

S5 Table Changes in the expression of genes adjacent to *parS* sequences and other ParB-binding sites in response to ParB overproduction. Data indicate the mean level of transcript in PAO1161 (pKGB9 *araBAD*p-*parB*) grown with (ParB+++) or without (ParB+) arabinose relative to PAO1161(pKGB8 *araBAD*) grown with 0.02% arabinose (EV) as revealed by the microarray analysis. The significance of the differences in expression (ANOVA) is indicated as *p*-value. Start / end for the indicated genes and ParB binding sites (*parS*s, peaks) refers to the genomic coordinates in PAO1 genome.
(XLSX)

S6 Table Genes with altered expression in response to ParB deficiency (PAO1161 *parB*_null_) and ParB overproduction [PAO1161 (pKGB9 *araBAD*p-*parB*) grown with or without 0.02% arabinose *vs* PAO1161 (pKGB8 *araBAD*p)]. (XLSX)

## References

[1] Salje J. Plasmid segregation: how to survive as an extra piece of DNA. Crit Rev Biochem Mol Biol. 2010;45: 296–317. doi: 10.3109/10409238.2010.494657 20586577 10.3109/10409238.2010.494657

[2] Baxter JC, Funnell BE. Plasmid partition mechanisms. Microbiol Spectr. 2014;2 doi: 10.1128/microbiolspec.PLAS-0023-2014 26104442 10.1128/microbiolspec.PLAS-0023-2014

[3] Gerdes K, Howard M, Szardenings F. Pushing and pulling in prokaryotic DNA segregation. Cell. 2010;141: 927–942. doi: 10.1016/j.cell.2010.05.033 20550930 10.1016/j.cell.2010.05.033

[4] Gerdes K, Møller-Jensen J, Jensen RB. Plasmid and chromosome partitioning: surprises from phylogeny. Mol Microbiol. 2000;37: 455–466. doi: 10.1046/j.1365-2958.2000.01975.x 10931339 10.1046/j.1365-2958.2000.01975.x

[5] Funnell BE. ParB partition proteins: complex formation and spreading at bacterial and plasmid centromeres. Mol Recognit. 2016; 44 doi: 10.3389/fmolb.2016.00044 10.3389/fmolb.2016.00044 PMC5002424 27622187

[6] Yamaichi Y, Niki H. Active segregation by the *Bacillus subtilis* partitioning system in *Escherichia coli*. Proc Natl Acad Sci. 2000;97: 14656–14661. doi: 10.1073/pnas.97.26.14656 11121066 10.1073/pnas.97.26.14656 PMC18974

[7] Livny J, Yamaichi Y, Waldor MK. Distribution of centromere-like *parS* sites in bacteria: insights from comparative genomics. J Bacteriol. 2007;189: 8693–8703. doi: 10.1128/JB.01239-07 17905987 10.1128/JB.01239-07 PMC2168934

[8] Mohl DA, Gober JW. Cell cycle–dependent polar localization of chromosome partitioning proteins in *Caulobacter crescentus*. Cell. 1997;88: 675–684. doi: 10.1016/S0092-8674(00)81910-8 9054507 10.1016/s0092-8674(00)81910-8

[9] Iniesta AA. ParABS system in chromosome partitioning in the bacterium *Myxococcus xanthus*. PLOS ONE. 2014;9: e86897 doi: 10.1371/journal.pone.0086897 24466283 10.1371/journal.pone.0086897 PMC3899335

[10] Ireton K, Gunther NW, Grossman AD. Spo0J is required for normal chromosome segregation as well as the initiation of sporulation in *Bacillus subtilis*. J Bacteriol. 1994;176: 5320–5329. 8071208 10.1128/jb.176.17.5320-5329.1994 PMC196717

[11] Sharpe ME, Errington J. The *Bacillus subtilis soj*-*spo0J* locus is required for a centromere-like function involved in prespore chromosome partitioning. Mol Microbiol. 1996;21: 501–509. doi: 10.1111/j.1365-2958.1996.tb02559.x 8866474 10.1111/j.1365-2958.1996.tb02559.x

[12] Lewis PJ, Errington J. Direct evidence for active segregation of *oriC* regions of the *Bacillus subtilis* chromosome and co-localization with the Spo0J partitioning protein. Mol Microbiol. 1997;25: 945–954. 9364919 10.1111/j.1365-2958.1997.mmi530.x

[13] Lin DC-H, Grossman AD. Identification and characterization of a bacterial chromosome partitioning site. Cell. 1998;92: 675–685. doi: 10.1016/S0092-8674(00)81135-6 9506522 10.1016/s0092-8674(00)81135-6

[14] Kim HJ, Calcutt MJ, Schmidt FJ, Chater KF. Partitioning of the linear chromosome during sporulation of *Streptomyces coelicolor* A3(2) involves an *oriC*-linked *parAB* locus. J Bacteriol. 2000;182: 1313–1320. 10671452 10.1128/jb.182.5.1313-1320.2000 PMC94417

[15] Fogel MA, Waldor MK. A dynamic, mitotic-like mechanism for bacterial chromosome segregation. Genes Dev. 2006;20: 3269–3282. doi: 10.1101/gad.1496506 17158745 10.1101/gad.1496506 PMC1686604

[16] Saint-Dic D, Frushour BP, Kehrl JH, Kahng LS. A parA homolog selectively influences positioning of the large chromosome origin in *Vibrio cholerae*. J Bacteriol. 2006;188: 5626–5631. doi: 10.1128/JB.00250-06 16855253 10.1128/JB.00250-06 PMC1540020

[17] Bartosik AA, Mierzejewska J, Thomas CM, Jagura-Burdzy G. ParB deficiency in *Pseudomonas aeruginosa* destabilizes the partner protein ParA and affects a variety of physiological parameters. Microbiology. 2009;155: 1080–1092. doi: 10.1099/mic.0.024661-0 19332810 10.1099/mic.0.024661-0 PMC2895232

[18] Lasocki K, Bartosik AA, Mierzejewska J, Thomas CM, Jagura-Burdzy G. Deletion of the *parA* (*soj*) homologue in *Pseudomonas aeruginosa* causes ParB instability and affects growth rate, chromosome segregation, and motility. J Bacteriol. 2007;189: 5762–5772. doi: 10.1128/JB.00371-07 17545287 10.1128/JB.00371-07 PMC1951838

[19] Vallet-Gely I, Boccard F. Chromosomal organization and segregation in *Pseudomonas aeruginosa*. PLOS Genet. 2013;9: e1003492 doi: 10.1371/journal.pgen.1003492 23658532 10.1371/journal.pgen.1003492 PMC3642087

[20] Gruber S, Errington J. Recruitment of condensin to replication origin regions by ParB/Spo0J promotes chromosome segregation in *B*. *subtilis*. Cell. 2009;137: 685–696. doi: 10.1016/j.cell.2009.02.035 19450516 10.1016/j.cell.2009.02.035

[21] Sullivan NL, Marquis KA, Rudner DZ. Recruitment of SMC by ParB-*parS* organizes the origin region and promotes efficient chromosome segregation. Cell. 2009;137: 697–707. doi: 10.1016/j.cell.2009.04.044 19450517 10.1016/j.cell.2009.04.044 PMC2892783

[22] Minnen A, Attaiech L, Thon M, Gruber S, Veening J-W. SMC is recruited to *oriC* by ParB and promotes chromosome segregation in *Streptococcus pneumoniae*. Mol Microbiol. 2011;81: 676–688. doi: 10.1111/j.1365-2958.2011.07722.x 21651626 10.1111/j.1365-2958.2011.07722.x

[23] Lee PS, Grossman AD. The chromosome partitioning proteins Soj (ParA) and Spo0J (ParB) contribute to accurate chromosome partitioning, separation of replicated sister origins, and regulation of replication initiation in *Bacillus subtilis*. Mol Microbiol. 2006;60: 853–869. doi: 10.1111/j.1365-2958.2006.05140.x 16677298 10.1111/j.1365-2958.2006.05140.x

[24] Murray H, Errington J. Dynamic control of the DNA replication initiation protein DnaA by Soj/ParA. Cell. 2008;135: 74–84. doi: 10.1016/j.cell.2008.07.044 18854156 10.1016/j.cell.2008.07.044

[25] Scholefield G, Whiting R, Errington J, Murray H. Spo0J regulates the oligomeric state of Soj to trigger its switch from an activator to an inhibitor of DNA replication initiation. Mol Microbiol. 2011;79: 1089–1100. doi: 10.1111/j.1365-2958.2010.07507.x 21235642 10.1111/j.1365-2958.2010.07507.x

[26] Figge RM, Easter J, Gober JW. Productive interaction between the chromosome partitioning proteins, ParA and ParB, is required for the progression of the cell cycle in *Caulobacter crescentus*. Mol Microbiol. 2003;47: 1225–1237. 12603730 10.1046/j.1365-2958.2003.03367.x

[27] Hajduk IV, Rodrigues CDA, Harry EJ. Connecting the dots of the bacterial cell cycle: coordinating chromosome replication and segregation with cell division. Semin Cell Dev Biol. 2016;53: 2–9. doi: 10.1016/j.semcdb.2015.11.012 26706151 10.1016/j.semcdb.2015.11.012

[28] Donczew M, Mackiewicz P, Wróbel A, Flärdh K, Zakrzewska-Czerwińska J, Jakimowicz D. ParA and ParB coordinate chromosome segregation with cell elongation and division during *Streptomyces sporulation*. Open Biol. 2016;6: 150263 doi: 10.1098/rsob.150263 27248800 10.1098/rsob.150263 PMC4852455

[29] Murray H, Ferreira H, Errington J. The bacterial chromosome segregation protein Spo0J spreads along DNA from *parS* nucleation sites. Mol Microbiol. 2006;61: 1352–1361. doi: 10.1111/j.1365-2958.2006.05316.x 16925562 10.1111/j.1365-2958.2006.05316.x

[30] Broedersz CP, Wang X, Meir Y, Loparo JJ, Rudner DZ, Wingreen NS. Condensation and localization of the partitioning protein ParB on the bacterial chromosome. Proc Natl Acad Sci. 2014;111: 8809–8814. doi: 10.1073/pnas.1402529111 24927534 10.1073/pnas.1402529111 PMC4066521

[31] Graham TGW, Wang X, Song D, Etson CM, Oijen AM van, Rudner DZ, et al ParB spreading requires DNA bridging. Genes Dev. 2014; doi: 10.1101/gad.242206.114 24829297 10.1101/gad.242206.114 PMC4052768

[32] Taylor JA, Pastrana CL, Butterer A, Pernstich C, Gwynn EJ, Sobott F, et al Specific and non-specific interactions of ParB with DNA: implications for chromosome segregation. Nucleic Acids Res. 2015;43: 719–731. doi: 10.1093/nar/gku1295 25572315 10.1093/nar/gku1295 PMC4333373

[33] Lagage V, Boccard F, Vallet-Gely I. Regional control of chromosome segregation in *Pseudomonas aeruginosa*. PLOS Genet. 2016;12: e1006428 doi: 10.1371/journal.pgen.1006428 27820816 10.1371/journal.pgen.1006428 PMC5098823

[34] Breier AM, Grossman AD. Whole-genome analysis of the chromosome partitioning and sporulation protein Spo0J (ParB) reveals spreading and origin-distal sites on the *Bacillus subtilis* chromosome. Mol Microbiol. 2007;64: 703–718. doi: 10.1111/j.1365-2958.2007.05690.x 17462018 10.1111/j.1365-2958.2007.05690.x

[35] Baek JH, Rajagopala SV, Chattoraj DK. Chromosome segregation proteins of *Vibrio cholerae* as transcription regulators. mBio. 2014;5: e01061–14. doi: 10.1128/mBio.01061-14 24803519 10.1128/mBio.01061-14 PMC4010829

[36] Attaiech L, Minnen A, Kjos M, Gruber S, Veening J-W. The ParB-*parS* chromosome segregation system modulates competence development in *Streptococcus pneumoniae*. mBio. 2015;6: e00662–15. doi: 10.1128/mBio.00662-15 26126852 10.1128/mBio.00662-15 PMC4488948

[37] Bartosik AA, Lasocki K, Mierzejewska J, Thomas CM, Jagura-Burdzy G. ParB of *Pseudomonas aeruginosa*: interactions with its partner ParA and its target *parS* and specific effects on bacterial growth. J Bacteriol. 2004;186: 6983–6998. doi: 10.1128/JB.186.20.6983-6998.2004 15466051 10.1128/JB.186.20.6983-6998.2004 PMC522188

[38] Jecz P, Bartosik AA, Glabski K, Jagura-Burdzy G. A Single *parS* Sequence from the cluster of four sites closest to *oriC* is necessary and sufficient for proper chromosome segregation in *Pseudomonas aeruginosa*. PLOS ONE. 2015;10: e0120867 doi: 10.1371/journal.pone.0120867 25794281 10.1371/journal.pone.0120867 PMC4368675

[39] Bartosik AA, Glabski K, Jecz P, Mikulska S, Fogtman A, Koblowska M, et al Transcriptional profiling of ParA and ParB Mutants in actively dividing cells of an opportunistic human pathogen *Pseudomonas aeruginosa*. PLOS ONE. 2014;9: e87276 doi: 10.1371/journal.pone.0087276 24498062 10.1371/journal.pone.0087276 PMC3909081

[40] Kusiak M, Gapczynska A, Plochocka D, Thomas CM, Jagura-Burdzy G. Binding and spreading of ParB on DNA determine its biological function in *Pseudomonas aeruginosa*. J Bacteriol. 2011;193: 3342–3355. doi: 10.1128/JB.00328-11 21531806 10.1128/JB.00328-11 PMC3133298

[41] Mierzejewska J, Bartosik AA, Macioszek M, Płochocka D, Thomas CM, Jagura-Burdzy G. Identification of C-terminal hydrophobic residues important for dimerization and all known functions of ParB of *Pseudomonas aeruginosa*. Microbiol Read Engl. 2012;158: 1183–1195. doi: 10.1099/mic.0.056234–0 10.1099/mic.0.056234-0 PMC3542827 22322962

[42] Miller JH. Experiments in molecular genetics 1972; Cold Spring Harbor Laboratory Press, Cold Spring Harbor, NY

[43] Hanahan D. Studies on transformation of *Escherichia coli* with plasmids. J Mol Biol. 1983;166: 557–580. doi: 10.1016/S0022-2836(83)80284-8 6345791 10.1016/s0022-2836(83)80284-8

[44] Simon R, Priefer U, Pühler A. A broad host range mobilization system for in vivo genetic engineering: transposon mutagenesis in gram negative bacteria. Nat Biotechnol. 1983;1: 784–791. doi: 10.1038/nbt1183-784

[45] Irani VR, Rowe JJ. Enhancement of transformation in *Pseudomonas aeruginosa* PAO1 by Mg2+ and heat. BioTechniques. 1997;22: 54–56. 8994645 10.2144/97221bm09

[46] Sambrook J, Fritsch EF, Maniatis T. Molecular cloning: a laboratory manual Cold Spring Harbor Laboratory; 1989.

[47] Ludwiczak M, Dolowy P, Markowska A, Szarlak J, Kulinska A, Jagura-Burdzy G. Global transcriptional regulator KorC coordinates expression of three backbone modules of the broad-host-range RA3 plasmid from IncU incompatibility group. Plasmid. 2013;70: 131–145. doi: 10.1016/j.plasmid.2013.03.007 23583562 10.1016/j.plasmid.2013.03.007

[48] El-Sayed AK, Hothersall J, Thomas CM. Quorum-sensing-dependent regulation of biosynthesis of the polyketide antibiotic mupirocin in *Pseudomonas fluorescens* NCIMB 10586. Microbiology. 2001;147: 2127–2139. doi: 10.1099/00221287-147-8-2127 11495990 10.1099/00221287-147-8-2127

[49] Guzman LM, Belin D, Carson MJ, Beckwith J. Tight regulation, modulation, and high-level expression by vectors containing the arabinose PBAD promoter. J Bacteriol. 1995;177: 4121–4130. 7608087 10.1128/jb.177.14.4121-4130.1995 PMC177145

[50] Marx CJ, Lidstrom ME. Development of improved versatile broad-host-range vectors for use in methylotrophs and other Gram-negative bacteria. Microbiol Read Engl. 2001;147: 2065–2075. doi: 10.1099/00221287-147-8-2065 11495985 10.1099/00221287-147-8-2065

[51] Reece KS, Phillips GJ. New plasmids carrying antibiotic-resistance cassettes. Gene. 1995;165: 141–142. doi: 10.1016/0378-1119(95)00529-F 7489905 10.1016/0378-1119(95)00529-f

[52] Soukas A, Cohen P, Socci ND, Friedman JM. Leptin-specific patterns of gene expression in white adipose tissue. Genes Dev. 2000;14: 963–980. doi: 10.1101/gad.14.8.963 10783168 PMC316534

[53] Saeed AI, Sharov V, White J, Li J, Liang W, Bhagabati N, et al TM4: a free, open-source system for microarray data management and analysis. BioTechniques. 2003;34: 374–378. 12613259 10.2144/03342mt01

[54] Pfaffl MW. A new mathematical model for relative quantification in real-time RT-PCR. Nucleic Acids Res. 2001;29: e45 11328886 10.1093/nar/29.9.e45 PMC55695

[55] O’Toole GA, Kolter R. Initiation of biofilm formation in *Pseudomonas fluorescens* WCS365 proceeds via multiple, convergent signalling pathways: a genetic analysis. Mol Microbiol. 1998;28: 449–461. doi: 10.1046/j.1365-2958.1998.00797.x 9632250 10.1046/j.1365-2958.1998.00797.x

[56] Kovach ME, Elzer PH, Hill DS, Robertson GT, Farris MA, Roop RM, et al Four new derivatives of the broad-host-range cloning vector pBBR1MCS, carrying different antibiotic-resistance cassettes. Gene. 1995;166: 175–176. 8529885 10.1016/0378-1119(95)00584-1

[57] Winsor GL, Lo R, Ho Sui SJ, Ung KSE, Huang S, Cheng D, et al *Pseudomonas aeruginosa* genome database and PseudoCAP: facilitating community-based, continually updated, genome annotation. Nucleic Acids Res. 2005;33: D338–343. doi: 10.1093/nar/gki047 15608211 10.1093/nar/gki047 PMC540001

[58] Yamamoto M, Ueda A, Kudo M, Matsuo Y, Fukushima J, Nakae T, et al Role of MexZ and PA5471 in transcriptional regulation of *mexXY* in *Pseudomonas aeruginosa*. Microbiology. 2009;155: 3312–3321. doi: 10.1099/mic.0.028993-0 19589837 10.1099/mic.0.028993-0

[59] Hay T, Fraud S, Lau CH-F, Gilmour C, Poole K. Antibiotic inducibility of the *mexXY* multidrug efflux operon of *Pseudomonas aeruginosa*: involvement of the MexZ anti-repressor ArmZ. PLoS ONE. 2013;8: e56858 doi: 10.1371/journal.pone.0056858 23441219 10.1371/journal.pone.0056858 PMC3575510

[60] Mac Aogáin M, Mooij MJ, McCarthy RR, Plower E, Wang YP, Tian ZX, et al The non-classical ArsR-family repressor PyeR (PA4354) modulates biofilm formation in *Pseudomonas aeruginosa*. Microbiol Read Engl. 2012;158: 2598–2609. doi: 10.1099/mic.0.058636–0 10.1099/mic.0.058636-0 22820840

[61] Hoffman LR, D’Argenio DA, MacCoss MJ, Zhang Z, Jones RA, Miller SI. Aminoglycoside antibiotics induce bacterial biofilm formation. Nature. 2005;436: 1171–1175. doi: 10.1038/nature03912 16121184 10.1038/nature03912

[62] Cirz RT, O’Neill BM, Hammond JA, Head SR, Romesberg FE. Defining the *Pseudomonas aeruginosa* SOS response and its role in the global response to the antibiotic ciprofloxacin. J Bacteriol. 2006;188: 7101–7110. doi: 10.1128/JB.00807-06 17015649 10.1128/JB.00807-06 PMC1636241

[63] McFarland KA, Dolben EL, LeRoux M, Kambara TK, Ramsey KM, Kirkpatrick RL, et al A self-lysis pathway that enhances the virulence of a pathogenic bacterium. Proc Natl Acad Sci. 2015;112: 8433–8438. doi: 10.1073/pnas.1506299112 26100878 10.1073/pnas.1506299112 PMC4500232

[64] Matsui H, Sano Y, Ishihara H, Shinomiya T. Regulation of pyocin genes in *Pseudomonas aeruginosa* by positive (*prtN*) and negative (*prtR*) regulatory genes. J Bacteriol. 1993;175: 1257–1263. 8444788 10.1128/jb.175.5.1257-1263.1993 PMC193209

[65] Hood RD, Singh P, Hsu F, Güvener T, Carl MA, Trinidad RRS, et al A type VI secretion system of *Pseudomonas aeruginosa* targets a toxin to bacteria. Cell Host Microbe. 2010;7: 25–37. doi: 10.1016/j.chom.2009.12.007 20114026 10.1016/j.chom.2009.12.007 PMC2831478

[66] Lesic B, Starkey M, He J, Hazan R, Rahme LG. Quorum sensing differentially regulates *Pseudomonas aeruginosa* type VI secretion locus I and homologous loci II and III, which are required for pathogenesis. Microbiol Read Engl. 2009;155: 2845–2855. doi: 10.1099/mic.0.029082–0 10.1099/mic.0.029082-0 PMC2888175 19497948

[67] Turner KH, Vallet-Gely I, Dove SL. Epigenetic control of virulence gene expression in *Pseudomonas aeruginosa* by a LysR-type transcription regulator. PLOS Genet. 2009;5: e1000779 doi: 10.1371/journal.pgen.1000779 20041030 10.1371/journal.pgen.1000779 PMC2796861

[68] Wade DS, Calfee MW, Rocha ER, Ling EA, Engstrom E, Coleman JP, et al Regulation of Pseudomonas quinolone signal synthesis in *Pseudomonas aeruginosa*. J Bacteriol. 2005;187: 4372–4380. doi: 10.1128/JB.187.13.4372-4380.2005 15968046 10.1128/JB.187.13.4372-4380.2005 PMC1151766

[69] Sams T, Baker Y, Hodgkinson J, Gross J, Spring D, Welch M. The Pseudomonas quinolone signal (PQS). Isr J Chem. 2016;56: 282–294. doi: 10.1002/ijch.201400128 10.1039/c5sc04197j PMC5477026 28660026

[70] Déziel E, Gopalan S, Tampakaki AP, Lépine F, Padfield KE, Saucier M, et al The contribution of MvfR to *Pseudomonas aeruginosa* pathogenesis and quorum sensing circuitry regulation: multiple quorum sensing-regulated genes are modulated without affecting *lasRI*, *rhlRI* or the production of N-acyl- l-homoserine lactones. Mol Microbiol. 2005;55: 998–1014. doi: 10.1111/j.1365-2958.2004.04448.x 15686549 10.1111/j.1365-2958.2004.04448.x

[71] Xiao G, He J, Rahme LG. Mutation analysis of the *Pseudomonas aeruginosa mvfR* and *pqsABCDE* gene promoters demonstrates complex quorum-sensing circuitry. Microbiol Read Engl. 2006;152: 1679–1686. doi: 10.1099/mic.0.28605–0 10.1099/mic.0.28605-0 16735731

[72] Zheng P, Sun J, Geffers R, Zeng A-P. Functional characterization of the gene *PA2384* in large-scale gene regulation in response to iron starvation in *Pseudomonas aeruginosa*. J Biotechnol. 2007;132: 342–352. doi: 10.1016/j.jbiotec.2007.08.013 17889392 10.1016/j.jbiotec.2007.08.013

[73] Mao X, Ma Q, Zhou C, Chen X, Zhang H, Yang J, et al DOOR 2.0: presenting operons and their functions through dynamic and integrated views. Nucleic Acids Res. 2014;42: D654–659. doi: 10.1093/nar/gkt1048 24214966 10.1093/nar/gkt1048 PMC3965076

[74] Solovyev V., Salamov A. Automatic annotation of microbial genomes and metagenomic sequences In: Li RW, editor. Metagenomics and its applications in agriculture, biomedicine and environmental studies. Nova Science Publishers; 2011 pp. 61–78.

[75] Wang X, Le TBK, Lajoie BR, Dekker J, Laub MT, Rudner DZ. Condensin promotes the juxtaposition of DNA flanking its loading site in *Bacillus subtilis*. Genes Dev. 2015;29: 1661–1675. doi: 10.1101/gad.265876.115 26253537 10.1101/gad.265876.115 PMC4536313

[76] Hittle LE, Powell DA, Jones JW, Tofigh M, Goodlett DR, Moskowitz SM, et al Site-specific activity of the acyltransferases HtrB1 and HtrB2 in *Pseudomonas aeruginosa* lipid A biosynthesis. Pathog Dis. 2015;73 doi: 10.1093/femspd/ftv053 26223882 10.1093/femspd/ftv053 PMC4626592

[77] Wang B, Li B, Liang Y, Li J, Gao L, Chen L, et al Pleiotropic effects of temperature-regulated 2-OH-lauroytransferase (PA0011) on *Pseudomonas aeruginosa* antibiotic resistance, virulence and type III secretion system. Microb Pathog. 2016;91: 5–17. doi: 10.1016/j.micpath.2015.11.003 26596709 10.1016/j.micpath.2015.11.003

[78] Shen L, Ma Y, Liang H. Characterization of a novel gene related to antibiotic susceptibility in *Pseudomonas aeruginosa*. J Antibiot (Tokyo). 2012;65: 59–65. doi: 10.1038/ja.2011.111 22146126 10.1038/ja.2011.111

[79] Liang Y, Guo Z, Gao L, Guo Q, Wang L, Han Y, et al The role of the temperature-regulated acyltransferase (PA3242) on growth, antibiotic resistance and virulence in *Pseudomonas aeruginosa*. Microb Pathog. 2016;101: 126–135. doi: 10.1016/j.micpath.2016.09.019 27746380 10.1016/j.micpath.2016.09.019

[80] Evensen G, Seeberg E. Adaptation to alkylation resistance involves the induction of a DNA glycosylase. Nature. 1982;296: 773–775. 7040984 10.1038/296773a0

[81] Hengge R. Principles of c-di-GMP signalling in bacteria. Nat Rev Microbiol. 2009;7: 263–273. doi: 10.1038/nrmicro2109 19287449 10.1038/nrmicro2109

[82] Valentini M, Filloux A. Biofilms and Cyclic di-GMP (c-di-GMP) Signaling: lessons from *Pseudomonas aeruginosa* and other bacteria. J Biol Chem. 2016;291: 12547–12555. doi: 10.1074/jbc.R115.711507 27129226 10.1074/jbc.R115.711507 PMC4933438

[83] Stover CK, Pham XQ, Erwin AL, Mizoguchi SD, Warrener P, Hickey MJ, et al Complete genome sequence of *Pseudomonas aeruginosa* PAO1, an opportunistic pathogen. Nature. 2000;406: 959–964. doi: 10.1038/35023079 10984043 10.1038/35023079

[84] Potvin E, Sanschagrin F, Levesque RC. Sigma factors in *Pseudomonas aeruginosa*. FEMS Microbiol Rev. 2008;32: 38–55. doi: 10.1111/j.1574-6976.2007.00092.x 18070067 10.1111/j.1574-6976.2007.00092.x

[85] Veening J-W, Smits WK, Kuipers OP. Bistability, epigenetics, and bet-hedging in bacteria. Annu Rev Microbiol. 2008;62: 193–210. doi: 10.1146/annurev.micro.62.081307.163002 18537474 10.1146/annurev.micro.62.081307.163002

[86] Penterman J, Singh PK, Walker GC. Biological cost of pyocin production during the SOS response in *Pseudomonas aeruginosa*. J Bacteriol. 2014;196: 3351–3359. doi: 10.1128/JB.01889-14 25022851 10.1128/JB.01889-14 PMC4135695

[87] Kreuzer KN. DNA damage responses in prokaryotes: regulating gene expression, modulating growth patterns, and manipulating replication forks. Cold Spring Harb Perspect Biol. 2013;5: a012674 doi: 10.1101/cshperspect.a012674 24097899 10.1101/cshperspect.a012674 PMC3809575

[88] Baharoglu Z, Mazel D. SOS, the formidable strategy of bacteria against aggressions. FEMS Microbiol Rev. 2014;38: 1126–1145. doi: 10.1111/1574-6976.12077 24923554 10.1111/1574-6976.12077

[89] Modell JW, Hopkins AC, Laub MT. A DNA damage checkpoint in *Caulobacter crescentus* inhibits cell division through a direct interaction with FtsW. Genes Dev. 2011;25: 1328–1343. doi: 10.1101/gad.2038911 21685367 10.1101/gad.2038911 PMC3127433

[90] Erental A, Kalderon Z, Saada A, Smith Y, Engelberg-Kulka H. Apoptosis-like death, an extreme SOS response in *Escherichia coli*. mBio. 2014;5: e01426–14. doi: 10.1128/mBio.01426-14 25028428 10.1128/mBio.01426-14 PMC4161249

[91] Nariya H, Inouye M. MazF, an mRNA interferase, mediates programmed cell death during multicellular *Myxococcus* development. Cell. 2008;132: 55–66. doi: 10.1016/j.cell.2007.11.044 18191220 10.1016/j.cell.2007.11.044

