# Supplementary figures and images for: Increased ParB level affects expression of stress response, adaptation and virulence operons and potentiates repression of promoters adjacent to the high affinity binding sites parS3 and parS4 in Pseudomonas aeruginosa
Source: PLoS One. 2017 Jul 21;12(7):e0181726. doi: 10.1371/journal.pone.0181726 (PMC5521831; doi:10.1371/journal.pone.0181726)

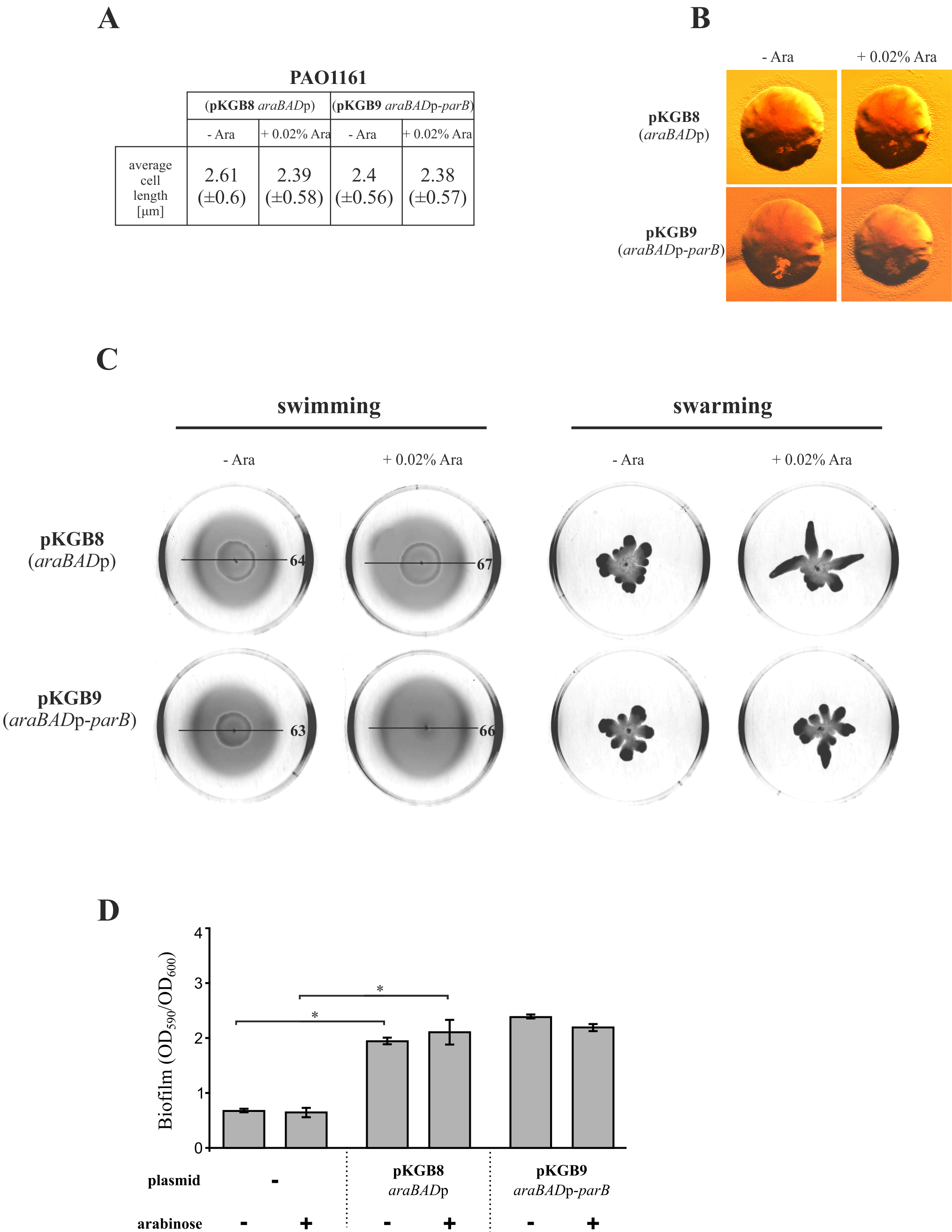

Supplement: S1 Fig — (A) Overnight cultures of PAO1161 (pKGB9) and PAO1161 (pKGB8 araBADp) were 100-fold diluted into L broth without and with 0.02% arabinose and grown to OD600 0.6. Cells were collected for microscopic observations, fixed and stained with DAPI (4,6-diamidino-2-phenylindole) as described previously1. Cells were observed using fluorescent microscope Carl Zeiss Axio Imager.M2 with appropriate lens EC Plan-Neofluar 100x/1.30 Oil Ph 3 M27 and camera AxioCamMR5. Collected pictures were analyzed with the program AxioVision Rel.4.8.2 (Carl Zeiss). The average cell length (±SD) in [μm] were calculated for at least 500 cells. (B) Colony morphology of PAO1161 (pKGB9) and PAO1161 (pKGB8) strains was observed using stereomicroscope Nikon SMZ1500 after 24 h incubation at 37°C on L agar plates with 75 μg ml-1 chloramphenicol with 0.02% arabinose or without. Images were captured with NIS-Elements 2.10 software. (C) Motility of PAO1161 (pKGB9) and PAO1161 (pKGB8) strains was analyzed as previously described1. Strains were grown overnight under selection and without or with 0.02% arabinose. All sets of plates for swimming and swarming tests were standardized by using the same volume of medium. The plates were inoculated with a sterile toothpick incubated for 48 h at 30°C and photographed. In the case of swimming plates, diameters of the swimming zones are indicated. (D) Biofilm formation in the overnight static cultures of PAO1161, PAO1161 (pKGB8) and PAO1161 (pKGB9). Strains were grown without or with 0.02% arabinose. Biofilm was stained with crystal violet and assessed by measurement of OD590. Data represent mean OD590/OD600 ratio ±SD from 3 biological replicates. *—p-value < 0.05 in two-sided Student’s t-test assuming equal variance.1 Lasocki et al., J Bacteriol. 2007;189: 5762–5772. 10.1128/JB.00371-07 (TIF) [file pone.0181726.s001.tif]

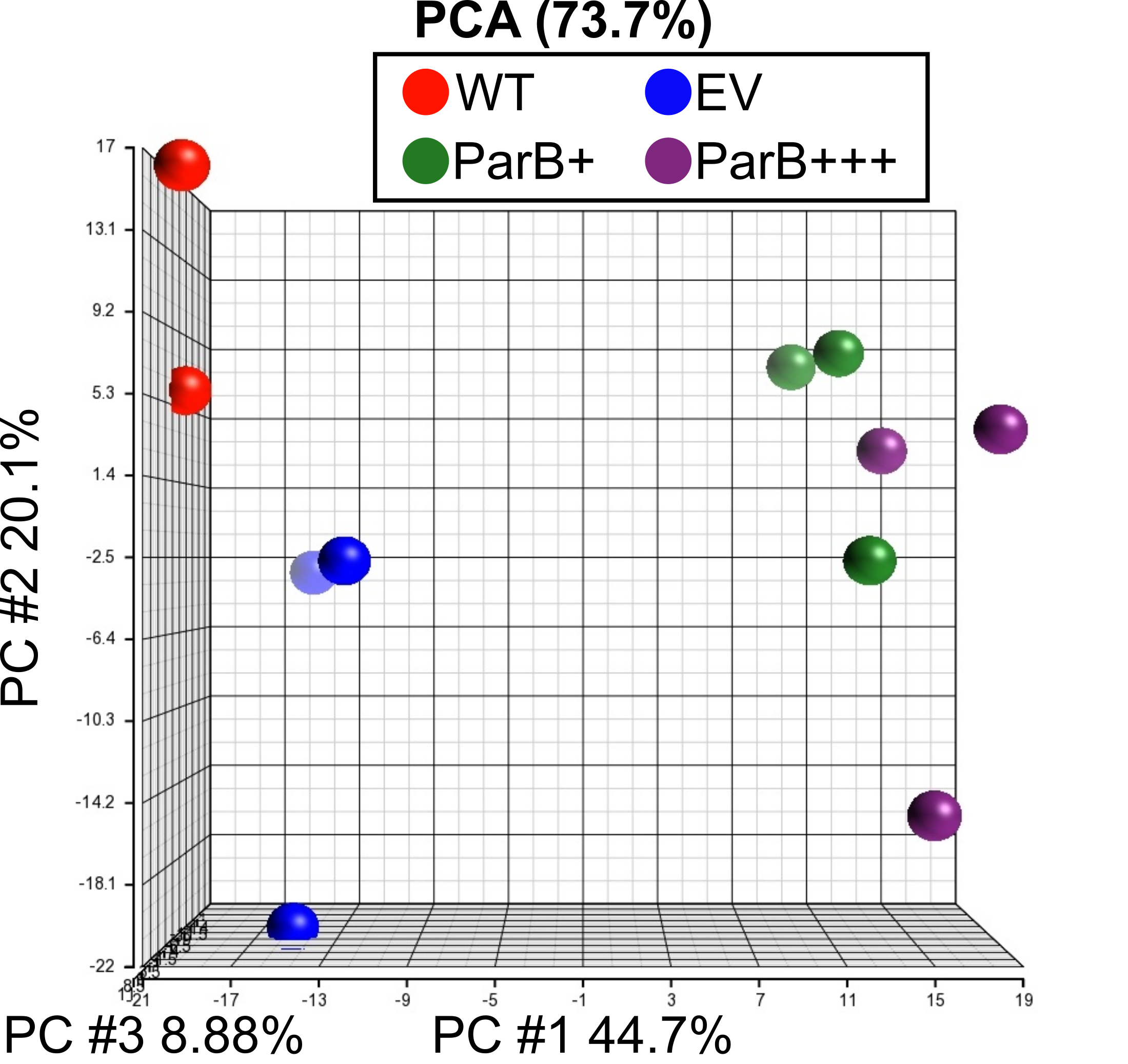

Supplement: S2 Fig — (TIF) [file pone.0181726.s002.tif]

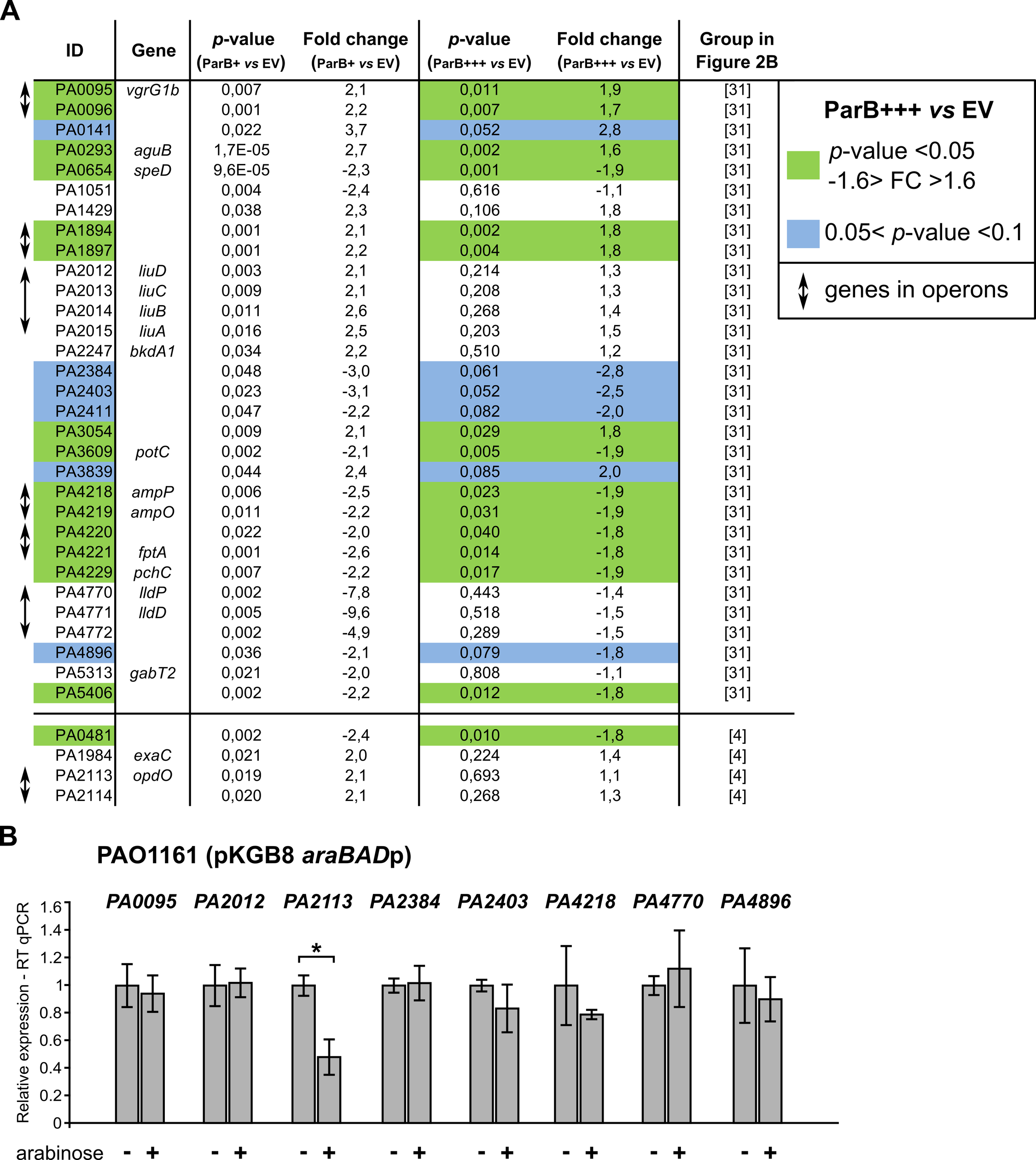

Supplement: S3 Fig — Abbreviation ParB+ corresponds to PAO1161 (pKGB9 araBADp-parB) strain grown without arabinose, EV corresponds to PAO1161 (pKGB8 araBADp) grown with arabinose and ParB+++ for PAO1161 (pKGB9 araBADp-parB) cells grown with arabinose. (A) Fold change- and p- values for the 35 genes in ParB+ vs EV and ParB+++ vs EV analysis. First subgroup of genes with -1.6> FC >-2 or 1.6< FC <2 and p-value <0.05 in ParB+++ vs EV comparison is indicated in green. The second subgroup of genes with 0.05< p-value <0.1 in ParB+++ vs EV comparison is indicated in blue. The third subgroup of genes with p-value >0.1 and -1.5< FC < 1.5 is left uncoloured. Genes in operons are marked with arrows. (B) Impact of arabinose on the expression of selected genes. PAO1161 (pKGB8 araBADp) cells were grown in L broth containing chloramphenicol with or without 0.02% arabinose. RT-qPCR was performed cDNA synthesized on RNA isolated from cells harvested at OD600 0.5. Data represent mean ±SD from three biological replicates. Expression values for all genes are shown relative to the cells from cultures without arabinose. *—p-value < 0.05 in two-sided Student’s t-test assuming equal variance. (TIF) [file pone.0181726.s003.tif]

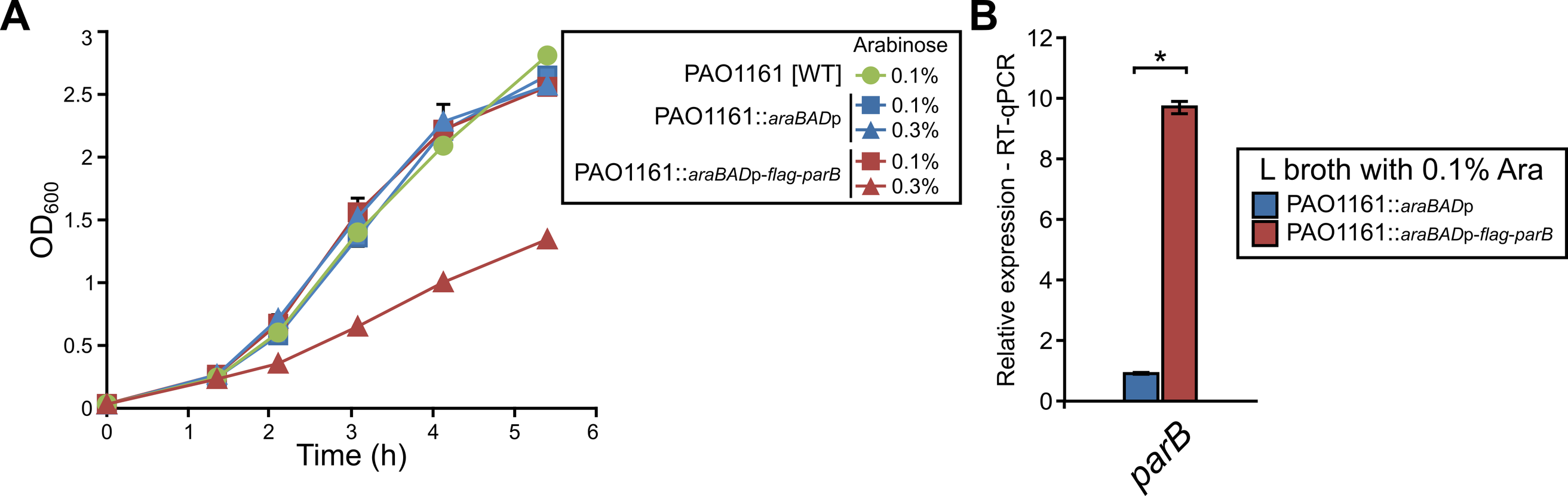

Supplement: S4 Fig — (A) Growth of P. aeruginosa PAO1161::araBADp, PAO1161::araBADp-flag-parB and PAO1161 strains in L broth with different arabinose concentrations. Data for PAO1161::araBADp-flag-parB grown on 0.1% arabinose represent mean OD600 for three biological replicates ±SD. (B) Impact of 0.1% arabinose on the expression of parB in PAO1161::araBADp-flag-parB relatively to PAO1161::araBADp cells. Strains were grown in L broth with 0.1% arabinose. RT-qPCR was performed on RNA isolated from cultures harvested at OD600 0.5. Data represent mean ±SD from three biological replicates. *—p-value < 0.05 in two-sided Student’s t-test assuming equal variance. (TIF) [file pone.0181726.s004.tif]
