# Supplementary material for: Increased ParB level affects expression of stress response, adaptation and virulence operons and potentiates repression of promoters adjacent to the high affinity binding sites parS3 and parS4 in Pseudomonas aeruginosa
Source: PLoS One. 2017 Jul 21;12(7):e0181726. doi: 10.1371/journal.pone.0181726 (PMC5521831; doi:10.1371/journal.pone.0181726)
Supplement: S1 Table — (DOCX) [file pone.0181726.s005.docx]

**Table S1**. Oligonucleotides used in this work.

| **Abb. Nr** | **Sequence 5´-3´** |
| --- | --- |
| **Primers for cloning** | |
| #1 | CATGGACTACAAGGACGACGATGACAAGGGCGGCGA |
| #2 | TATCGCCGCCCTTGTCATCGTCGTCCTTGTAGTC3 |
| #3 | TGACGCTTTTTATCGCAACTC |
| #4 | AGTTTGTAGAAATGCATAAAGGCCATCCGT |
| #5 | ACGGATGGCCTTTATGCATTTCTACAAACT |
| #6 | TCGTCGTTTGGTATGGCTTC |
| #7 | GTCAGAATTCATGGACTACAAGGACGACGA |
| #8 | TGCCATGAACTCGGATCCG |
| #9 | CGGATCCGAGTTCATGGCA |
| #10 | GTCAGTCGACTCAACGGATGTGGGCG |
| #11 | CTGACAATTGGTCCACATTCGGACTGGAAT |
| #12 | CTGACTGCAGGAGGTCAGTGTGCAGAGCCT |
| #13 | CGGTCTGCAGAACAGGAATTGGGGGAAATT |
| #14 | CTGAGGATCCGGCGATCGAAGCCTTG |
| #15 | TATAGCATGCGAATTCAAGGCCGGCTTCAACTTC |
| #16 | TATAGGATCCGAAACACTCCTCGCTCGACT |
| #17 | TATAGCATGCGAATTCCCAACAGGCAGTGTCGTTAC |
| #18 | TATAGGATCCGTGCTGTCCTTTTTGCGC |
| #19 | TATAATGCATGATGGTAGTGTGGGGTCTCC |
| #20 | CGGCTGATGTCCACAGTAGTTCACCACCT |
| #21 | TACTGTGGACATCAGCCGCTACAGTCAAC |
| #22 | GCTCACTCGAGCGCGAAATACGGGCAGACATGG |
| #23 | CGCGCTCGAGTGAGCAATAACTAGCATAACCCC |
| #24 | TATAATGCATGCCAATCCGGATATAGTTCCTCC |
| #25 | GGGAATTCCCCGCACCTCCAGGCAACG |
| #26 | GGGGATCCCGGTGGGCCCACGATACAG |
| **Primers used in qPCR analysis** | |
| **Name/gene ID** | **Sequence 5´-3´** |
| qPA0001F | TGCCGTCCCAACAATTCAAC |
| qPA0001R | AAACGGTTGGGTGCATACAC |
| qPA0002F | TGCAGCTTTCCAACGGTTTG |
| qPA0002R | TGTACTCGACCTGCACTTCTTC |
| qPA0003F | AGCATCGAATGGCTCTTTGC |
| qPA0003R | CCAGCCGTCTTTCAATAGTTGC |
| qPA0004F | TGCTCTGCTTCACCAACAAC |
| qPA0004R | TTGTTCAGGTTACGCGTCAG |
| qPA0005F | TACCGCTTCGTGGTACAGAAC |
| qPA0005R | TTTTCCGGGATGTTCTCCAGTC |
| qPA0006F | TATTACGACCTGGCAGTGCTC |
| qPA0006R | TGCGGACAATAGACGATGAGG |
| qPA0007F | TGCACCTTTCAAGCTTGTCC |
| qPA0007R | GCTTTCGGTCCATTCCTTGC |
| qPA0008F | TTGAACGAACTGCCGATTCC |
| qPA0008R | AACAGCATCACCAGCCATTG |
| qPA0009F | TACATCCTTCGCGTGCGTAC |
| qPA0009R | TACTTCGTCACGCAATTCGG |
| qPA0010F | AACGACGATCCCCTCTACATG |
| qPA0010R | AGCAGCAGAAGTTCGAACAG |
| qPA0011F | AAGTGAAGAAAGGCGGTTGC |
| qPA0011R | TGCTGATCAATGCAGTGGTG |
| qPA0012F | TTATTTGGCGAGCTGATGGC |
| qPA0012R | AGTAACGACACTGCCTGTTG |
| qPA0013F | TCATCATCATCGCCAGCAAC |
| qPA0013R | ATGAACGGAAAGCTGAAGGC |
| qPA0014F | TGAGATGCTCGAATCCGTACAG |
| qPA0014R | TCCGCACTTCGATCCTTTCC |
| qPA0015F | GTGGACAATGCGCTGCTG |
| qPA0015R | TACTTCGGGTCCTGCTCTAC |
| qPA0016F | TGCTGCTGGAGGAAAACATC |
| qPA0016R | AGCAGCGAGGACATGATGTTG |
| qPA0095F | ATCAGCGGCAAGAACATCAC |
| qPA0095R | TCAGTTCTGGAGGATCTTGCG |
| qPA0492F | TCGAGCTGGTCAACGACATG |
| qPA0492R | TTGACGTGTTGCAGCGACAG |
| qPA0493F | TTGCCCGGCACCTTCTATC |
| qPA0493R | ACTGCTTCATCACCTCGATCAG |
| qPA0494F | TGTACCTGGAGCGCTTCATC |
| qPA0494R | TCGCGTTCGAACAGATGCAC |
| qPA0612F | ATCACGCCAACGAACTGGTC |
| qPA0612R | CAGTCCTCGCAGTCTTCCG |
| qPA0985F | AAGTGCTTTGCCTCCATTGG |
| qPA0985R | TTGTTTGTGGTCTGCAGCTG |
| qPA0996F | TTCTGTTCCGCCTCGATTTC |
| qPA0996R | AGAGAATGTAGGTCCGGCATTG |
| qPA1003F | CGTACTGCTCGACGATTTCAAC |
| qPA1003R | TATCGATTTCCGCGTTGTCC |
| qPA2008F | TTCTGCCTGCTCAACGACTG |
| qPA2008R | TGAAGCTCTTCGACAGGAACG |
| qPA2012F | CGATGAACGGCAGTATCGTG |
| qPA2012R | GCTGTGTTCCATCTTCATCGC |
| qPA2113F | AAGGAATGGGAACGCAACAC |
| qPA2113R | TCGTTGGCGAAATTCGAACG |
| qPA2384F | CGTCTCTCCGAAACCGGTAC |
| qPA2384R | TGTAGACATCCTGTTCGTCGAG |
| PA2403F | CGACCCTGTACATCAACCCC |
| PA2403R | GTTGTTGAACGGAACCAGCC |
| qPA2432F | TATCGAAGCCTTCCTCAACGTC |
| qPA2432R | TGCTGATCACCGACTTGGAC |
| qPA3661F | AAGACCCGCAACCAGATGAG |
| qPA3661R | TCATGCTCTCGACGCTGTC |
| qPA4218F | TGGGTGGTCAAGTTCCTCTG |
| qPA4218R | CATCGGCAGGATCCAGCTAC |
| qPA4635F | ACATCCTGTTGCGCGACATC |
| qPA4635R | ATCTGCACTTCGTAGCGTTG |
| qPA4659F | TAGCGACCCTCTGCGAAAAG |
| qPA4659R | TGCGCAACCAGGAGAAGAAG |
| qPA4770F | ATGATCTCCCCGCAATCTATCG |
| qPA4770R | AAGATCAGGCTGTGCTTCAGG |
| qPA4896F | AACAACAGAGCGTGATCGTC |
| qPA4896R | TTCTGCTTCATGCCGTCCAG |
| qPA5471F | CGGCGGACAAACTGGAAAAG |
| qPA5471R | TAGATCCAGGGCTTCGATGC |
| qPA5562F | CAGATCCGGCATGGTCAGAA |
| qPA5562R | CAACGGATGTGGGCGAGAA |
| NADBF | CTACCTGGACATCAGCCACA |
| NADBR | GGTAATGTCGATGCCGAAGT |
