# Supplementary material for: Increased ParB level affects expression of stress response, adaptation and virulence operons and potentiates repression of promoters adjacent to the high affinity binding sites parS3 and parS4 in Pseudomonas aeruginosa
Source: PLoS One. 2017 Jul 21;12(7):e0181726. doi: 10.1371/journal.pone.0181726 (PMC5521831; doi:10.1371/journal.pone.0181726)
Supplement: S4 Table — Upper panel demonstrates the growth test of PAO1161 (pKGB8) and PAO1161 (pKGB9) in the presence of a range of antibiotic concentrations (2-fold dilution steps) relatively to the standard Pa strain ATCC 27853. The bottom panel demonstrates the range of concentrations tested for chosen antibiotics and EUCAST 2016 (European Committee on Antimicrobial Susceptibility) breakpoint table for interpretation of MICs. Cells were grown in Mueller Hinton cation adjusted broth (Difco) supplemented with antibiotics and arabinose (Ara) as indicated. Chloramphenicol (Cm) at concentration of 75 µg ml-1 was added to maintain the plasmids. (DOCX) [file pone.0181726.s008.docx]

**S4 Table.** Influence of ParB overproduction on the antibiotic resistance of PAO1161 derivatives.

| **Strain** | **MHCA additives** | **piperacillin** | | **ticarcillin** | | **gentamicin** | | **ciprofloxacin** | | **imipenem** | | **tobramycin** | |
| --- | --- | --- | --- | --- | --- | --- | --- | --- | --- | --- | --- | --- | --- |
| **PAO1161** | 0.2% Ara | 8 | S | 32 | R | 2 | S | 0.125 | S | 1.5 | S | 1 | S |
|  | 0.02% Ara | 8 | S | 32 | R | 2 | S | 0.125 | S | 1 | S | 1.5 | S |
|  | - | 8 | S | 32 | R | 4 | S | 0.25 | S | 1 | S | 1 | S |
| **PAO1161 pKGB8 (*araBAD*p)** | 0.2% Ara, 75µg/ml Cm | 8 | S | 32 | R | 4 | S | 0.125 | S | 1 | S | 2 | S |
|  | 0.02% Ara, 75µg/ml Cm | 8 | S | 32 | R | 4 | S | 0.125 | S | 1 | S | 1.5 | S |
|  | 75µg/ml Cm | 8 | S | 32 | R | 4 | S | 0.125 | S | 1 | S | 2 | S |
| **PAO1161 pKGB9 (*araBAD*p-*parB*)** | 0.2% Ara, 75µg/ml Cm | 8 | S | 16 | S | 2 | S | 0.125 | S | 0.75 | S | 2 | S |
|  | 0.02% Ara, 75µg/ml Cm | 8 | S | 32 | R | 2 | S | 0.06 | S | 1 | S | 3 | S |
|  | 75µg/ml Cm | 8 | S | 32 | R | 4 | S | 0.125 | S | 1 | S | 3 | S |
| ***P. aeruginosa* ATCC 27853** | - | 8 | S | 16 | S | 2 | S | 0.25 | S | 1 | S | 0.75 | S |

| **Antibiotic** | **Range of concentrations tested** | **EUCAST 2016 sensitive (S), resistant (R)** |
| --- | --- | --- |
| piperacillin | 256-0.25 μg/ml | S≤16; R>16 |
| ticarcillin | 256-0.25 μg/ml | S≤16; R>16 |
| gentamicin | 64-0.06 μg/ml | S≤4; R>4 |
| ciprofloxacin | 32-0.03 μg/ml | S≤0.5; R>1 |
| imipenem | 32-0.002 μg/ml | S≤4; R>8 |
| tobramycin | 256-0.016 μg/ml | S≤4; R>4 |
